# Analytical and clinical validation of a high accuracy fully automated digital immunoassay for plasma phospho-Tau 217 for clinical use in detecting amyloid pathology

**DOI:** 10.3389/fneur.2025.1568971

**Published:** 2025-07-09

**Authors:** David Wilson, Meenakshi Khare, Gallen Triana-Baltzer, Michele Wolfe, Patrick Sheehy, Karen Copeland, Lyndal Hesterberg, Ann-Jeanette Vasko, Wiesje M. van der Flier, Inge M. W. Verberk, Charlotte E. Teunissen, Mike Miller

**Affiliations:** ^1^Quanterix Corporation, Billerica, MA, United States; ^2^Johnson and Johnson Innovative Medicine, La Jolla, CA, United States; ^3^Boulder Statistics, Steamboat Springs, CO, United States; ^4^HCS Control Systems, Denver, CO, United States; ^5^Neurochemistry Laboratory, Department of Laboratory Medicine, Amsterdam UMC, Vrije Universiteit Amsterdam, Amsterdam Neuroscience, Amsterdam, Netherlands

**Keywords:** Alzheimer’s disease, immunoassay, p-Tau 217, validation, amyloid, Simoa

## Abstract

**Background:**

With the emergence of disease-modifying therapies for Alzheimer’s disease (AD), there is an urgent need for scalable, accurate, and well-validated blood test alternatives to positron emission topography (PET) and lumbar punctures for identifying amyloid pathology to facilitate identification of candidates for therapy. Plasma p-Tau 217 has emerged as a plasma-based biomarker with sufficient sensitivity and specificity to both rule out and rule in amyloid pathology with high confidence, potentially serving as a readily scalable non-invasive test to aid AD diagnosis. In this report, we describe robust analytical and clinical validation of a lab developed test for plasma p-Tau 217 suitable for clinical diagnostic use.

**Methods:**

A high sensitivity digital immunoassay using single molecule array (Simoa) technology was developed for plasma p-Tau 217 utilizing a 2-cutoff approach. The assay was analytically validated with industry standard protocols and clinically validated across 873 symptomatic individuals from two independent clinical cohorts using PET or cerebrospinal fluid (CSF) biomarkers as comparators.

**Results:**

The assay exhibited acceptable analytical characteristics with an analytical sensitivity enabling measurement of plasma p-Tau 217 in all clinical samples. Excluding results between the two cutoffs, clinical sensitivity, specificity, and agreement with comparator methods (accuracy) were >90%, with 30.9% of the samples falling in the intermediate zone between the two cutoffs.

**Discussion:**

The performance characteristics of the Simoa p-Tau 217 assay align with current accuracy recommendations for blood-based biomarker test performance for diagnostic use, making the test suitable for clinical use under the Clinical Laboratory Improvement Act (CLIA) as a diagnostic plasma test to aid in Alzheimer’s diagnosis.

## 1 Introduction

FDA approval of disease-modifying treatments (DMT) for Alzheimer’s disease (AD) and the likelihood of other potential approved DMTs in the pipeline highlights the urgent need for non-invasive widely available blood tests to facilitate timely diagnosis toward identifying patients eligible for treatment. Currently established biomarker-based approaches to diagnostic workup include positron emission tomography (PET) imaging and cerebrospinal fluid (CSF) biomarkers for amyloid and phosphorylated tau, both of which are invasive, expensive, and may not be widely available. Fortunately, significant advances have been made in recent years in the development of blood tests for detecting AD pathology driven by advances in high-sensitivity laboratory methods and high-quality antibody reagents. One such method, single molecule array (Simoa), enabled the introduction of high sensitivity immunoassays for numerous blood-based biomarkers of relevance in AD research and potentially diagnostics, including Amyloid beta42/40 (Aβ42/40), phosphorylated-tau isoforms, glial fibrillary acidic protein (GFAP) and neurofilament light (NfL) (1). Among this slate of Simoa assays for plasma biomarkers, a high sensitivity assay for tau phosphorylated at the 217 residue (p-Tau 217) was described by Janssen R&D several years ago (2). This digital assay differs from less sensitive conventional chemiluminescence or electrochemiluminescence methods employing analog detection principles. The Simoa p-Tau 217 assay was employed in numerous studies that have contributed to an important body of evidence highlighting this p-Tau isoform as generally outperforming two other well studied isoforms (p-Tau 181 and p-Tau 231) for detection of amyloid and tau pathology (3) and longitudinal monitoring and prognosis of disease progression (4). Based on a sizable body of consistent evidence, a consensus has emerged that plasma p-Tau 217 represents the best single blood-based biomarker target currently available to aid in Alzheimer’s pathology detection. Reflecting this consensus, the Alzheimer’s Association (AA) Workgroup recently recommended plasma p-Tau 217 as the only blood-based biomarker that has demonstrated accuracy comparable to FDA-cleared CSF biomarker tests, enabling a confirmatory diagnostic use case with appropriate validation (5). Such a use case has the potential to significantly attenuate reliance on PET and lumbar punctures for the AD diagnostic pathway. The proposed AA criteria also recommends that a blood test for plasma p-Tau 217 be designed with two cut-offs in recognition of signal overlap between diseased and non-diseased patients. The use of two cutoffs maximizes the negative and positive predictive values of the test and yields a diagnostic ‘gray zone’ in which there is less certainty of amyloid status. The AA further recommended the plasma test should exhibit an accuracy of ≥90% for diagnostic use. Following closely behind the development of the AA guidance, the Us Against Alzheimer’s Global CEO initiative (CEOi) (6) similarly arrived at recommendations on confirmatory plasma test performance criteria and a 2-cutoff approach that mirrors that of the AA (7).

To address the need for a scalable high-accuracy blood test to facilitate AD diagnosis, we endeavored to validate a Simoa p-Tau 217 assay in accordance with CLIA standards and with sufficient clinical diversity and powering to establish robust diagnostic cutoffs for a lab developed test (LDT) for clinical use that meets AA and CEOi guidance for clinical performance. This report describes the analytical and clinical validation of this Simoa plasma p-Tau 217 test, branded “LucentAD p-Tau 217” to indicate its commercial availability for clinical use.

## 2 Methods

### 2.1 Apparatus

All Simoa p-Tau 217 assay testing was performed on the Simoa HD-X instrument, a fully automated digital immunoassay analyzer utilizing Simoa technology for isolation and counting of single molecules. The instrument pipettes sample directly from sample tubes or 96-well plates and processes immunoassays and data reduction with a steady state throughput of 66 tests/h. Details of the instrument and its principles are given elsewhere (8).

### 2.2 Assay principle and protocol

The single-molecule sensitivity of Simoa technology has been discussed (9). In brief, Simoa is a digitized bead-based enzyme linked immunosorbent assay (ELISA) whereby diffusion of fluorescent reporter molecules at the signal step is constrained to 40-femtoliter wells in a microarray. By restricting diffusion to such a small volume, fluorophores generated by a single enzyme label can be detected in the array in 30 s. The arrays are composed of 216,000 wells which are counted simultaneously. Simultaneous counting of all femto wells enables rapid signal acquisition leading to rapid assays, which generally take 45–60 min for complete processing. Through this simple digital processing approach, attomolar sensitivity can be obtained (9).

The Simoa p-Tau 217 assay design has been described (2). In brief, the assay is a 3-step sandwich immunoassay in which sample is drawn from the sample tube by the instrument pipettor and mixed with anti-p-Tau 217 coated paramagnetic capture beads in a reaction cuvette. Following collection of the beads with a magnet, washing, and redispersion, biotinylated detector antibodies are combined with the beads and incubated. Following a second bead collection and wash, a conjugate of streptavidin-ß-galactosidase (SβG) is mixed with the capture beads for the third assay step. Following a third bead collection and wash, the capture beads are resuspended in a resorufin ß-D-galactopyranoside (RGP) substrate solution. Digital processing occurs when beads are transferred to the Simoa array disc (10). Individual capture beads are sealed within microwells in the array. If p-Tau 217 has been captured and labeled, the ß-galactosidase hydrolyzes the RGP substrate into a fluorescent product that provides the signal for measurement. The concentration of p-Tau 217 in unknown samples is interpolated from a logistic 4-parameter standard curve. Time to assay completion per measurement is about an hour.

### 2.3 Reagents

Four reagents were developed for the assay: paramagnetic p-Tau 217 capture beads, biotinylated detector, SβG conjugate, and sample diluent. The capture beads comprised a monoclonal anti-p-Tau 217 antibody [Janssen PT3, (11)] specific for an epitope spanning residues 210–220 with two phosphorylation sites (212 and 217) covalently attached by standard coupling chemistry to 2.7 μm carboxy paramagnetic microbeads (Agilent Technologies). The antibody-coated beads were diluted in Tris buffer with a surfactant and protein stabilizer (bovine). Biotinylated detector reagent comprised a monoclonal anti-tau antibody (Janssen HT43) specific for N terminal residues 7–20 that was biotinylated using standard methods and diluted in a PBS diluent containing surfactant and BSA. SβG was prepared by covalent conjugation of purified streptavidin (Thermo Scientific) and βG (Sigma) using standard coupling chemistry and in a phosphate buffer with a surfactant and protein stabilizer (bovine). Sample Diluent was formulated in PBS diluent with heterophilic blockers, EDTA, and a surfactant.

The quality and functional performance of assay reagents, including bead coupling efficiency, detector antibody biotinylation, and SβG enzymatic activity, are routinely monitored as part of quality control processes. These procedures involve testing with released kits and endogenous control samples to ensure reagent consistency and assay robustness over time. All reagents used in this study met predefined acceptance criteria based on standard operating procedures designed to ensure reliable assay performance. Additionally, for key reagent components such as heterophilic blockers, rigorous incoming material quality control processes ensure that predefined performance specifications which may include functional testing are met before acceptance.

### 2.4 Calibration

The assay is calibrated using purified peptide construct (MW 4929, New England Peptide, Gardner MA) composed of the N-terminal epitope (tau residues 7–20) and mid-region phosphorylated epitope (tau residues 210–220, phosphorylated at 212 and 217), connected by a 4-unit polyethylene glycol linker (12). The peptides were HPLC purified, confirmed by mass spectral analysis, and the purified peptide mass-based concentration was determined by the manufacturer. Calibrators were prepared gravimetrically with nominal values of 0.002, 0.010, 0.039, 0.156, 0.625, 2.50, and 10.0 pg/mL based on volumetric dilutions and stored in phosphate buffer with a protein stabilizer (bovine), a surfactant, and ProClin 300 as a preservative.

### 2.5 Analytical verification

Key assay analytical performance characteristics were verified at the Quanterix CLIA laboratory (Billerica, MA, USA) in accordance with standard Clinical and Laboratory Standards Institute (CLSI) protocols. These studies verified performance across multiple instruments and reagent lots as indicated in Results.

#### 2.5.1 Linearity

Assay linearity was tested according to CLSI Document EP06 Ed2 (13) with three replicates of 10 K2EDTA plasma samples distributed across the assay range to within 10% of the upper reportable limit. The 10 samples were prepared by admixing contrived elevated plasma samples with a native low p-Tau 217 plasma pool to arrive at evenly spaced p-Tau 217 concentrations across the range. Linearity was evaluated by linear regression analysis. Dilution linearity was also assessed by serially diluting five native K2EDTA samples with high p-Tau 217 levels (upper third of assay range) using the Sample Diluent.

#### 2.5.2 Sensitivity

Detection capability for limit of blank (LoB), limit of detection (LoD), and lower limit of quantitation (LLoQ) was estimated in accordance with CLSI Document EP17-A2 (14) across two reagent lots and a single HD-X instrument. For LoB, 20 replicates of the zero calibrator were assessed for each lot of reagents. LoB was estimated with the non-parametric analysis method across the two lots as prescribed in CLSI EP17-A2.

For LoD, 3 native plasma samples with low levels of p-Tau 217 and 3 contrived samples prepared by spiking antigen into the zero calibrator at low levels were tested in duplicate across 2 reagent lots. The pooled standard deviation (SD_L_) across the low-level samples was calculated according to EP17-A2 where LOD = LOB + Cp × SD_L_, where the multiplier Cp is given by


$$\mathrm{Cp}=1.645/[1–(1/(4^{*}(L-J))]$$

Here L = total number of all low-level sample results across all reagents and J = number of low-level samples.

For LLoQ, a set of 18 native plasma samples from healthy donors expected to have concentrations near the anticipated LLoQ were tested in duplicate over two runs, each with a different lot of reagents. For each lot, the precision profile (p-Tau 217 vs. replicate CV) was evaluated for the point at which the non-linear fit crossed the 20% CV level to define the LLoQ for that lot. The LLoQ was based on the worst performing lot of the 2 lots tested. Measurement accuracy was verified by confirming that the back calculation of the lowest p-Tau 217 calibrator (0.002 pg/mL, less than all the native samples) was within 80–120% of the expected concentration.

#### 2.5.3 Repeatability and reproducibility

Repeatability and within-laboratory precision were assessed according to CLSI document EP05-A3 (15) using 5 native plasma samples and a 5-day × 2 run × 2 replicate design across 2 reagent lots, 2 instruments, and 2 analysts (20 replicates/instrument, 40 total replicates). The 5 samples approximated low (near LLoQ), medium, and high levels of the assay measuring range. Two contrived plasma quality control samples (low and high, contrived with CSF from Alzheimer’s patients spiked into plasma) were also tested. Intra-assay repeatability was tested with 5 K2EDTA plasma samples from presumed normal donors, tested in replicates of 20 each. The average CV for each sample was then evaluated.

#### 2.5.4 Specificity

Specificity of the assay was evaluated using synthetic tau peptides (Genscript Biotech, Piscataway, NJ, USA) which included the N-terminal epitope and phosphorylation site epitopes at one of the following amino acid residues: 181, 205, 212/217 (positive control), 231, 231/235. Each peptide was prepared at 0.03, 0.3, 3.0, and 30 pg/mL in calibrator diluent and tested in replicates of three with one reagent lot and one instrument. Un-spiked buffer was used as a negative control.

#### 2.5.5 Endogenous interferences

Interference testing was performed according to CLSI EP07-Ed3 (16). Three native K2EDTA plasma samples (one low, moderately positive, and one high positive, 0.024, 0.060, and 0.114 pg/mL respectively) were assessed for the impact of endogenous interferents (bilirubin, triglycerides, etc.) using one reagent lot and one instrument. Interferent stock solutions were prepared, where possible, at concentrations of at least 20 times the intended test concentration. Interferent stock solution was added to the test sample at a ratio of 1-part spiking solution stock to 19-parts sample. Equal volume of solvent used for the stock spiking solution (without interferent) was added to the control sample and care was taken not to dilute the matrix volume by more than 5%. In the case of total protein (human plasma albumin), the required amounts were directly weighed and added to the plasma samples. In case of human anti-mouse IgG (HAMA), a highly concentrated source was procured and diluted into the sample matrix to achieve the target concentration. All samples were tested in duplicate, and all test and corresponding control conditions were performed in the same assay.

#### 2.5.6 Sample stability

The stability of 6 native K2EDTA plasma specimens (range: 0.024–0.114 pg/mL) was assessed at room temperature, refrigerated (2–8°C) and after 3 freeze–thaw cycles using one reagent lot and one instrument with guidance from CLSI document EP25-A Vol. 29 No. 20 (17). Room temperature storage intervals were 4 and 8 h, and the refrigerated storage intervals were 24 and 48 h. −70°C storage served as the control condition.

#### 2.5.7 Analytical samples and other materials

To establish the detection capabilities of the p-tau 217 assay at the high end of the assay range where very high plasma p-Tau 217 levels are rare, CSF (50 pg/mL) from AD patients was used as a spiker into native K2EDTA samples. Similarly, endogenous quality control samples were prepared by spiking CSF from AD patients into commercially obtained K2EDTA plasma from individual presumed healthy donors. The use of p-Tau 217 from CSF (typically 10–100 times more concentrated than in plasma) is acknowledged to modify the plasma matrix, potentially affecting results compared to 100% plasma. The volumes of CSF were limited to a maximum of 1:20 (95% plasma) in order to minimize any matrix affects.

### 2.6 Clinical validation

Clinical performance for classifying amyloid status was validated by comparison with either CSF biomarkers or amyloid PET across two independent cohorts: the Amsterdam Dementia Cohort (ADC) (18, 19) and the Bio-Hermes cohort (20). Both cohorts were reviewed and approved by central or local ethics and safety review committees or boards. All participants (or their legally authorized representative) reviewed and signed an approved informed consent document to use medical data and biomaterials for research purposes.

#### 2.6.1 Amsterdam dementia cohort

The ADC represents all patients who present to the Alzheimer Center Amsterdam of the Amsterdam University Medical Centers. These patients were referred for analysis of their cognitive complaints by their general practitioner or their local specialist. Each patient received the same standardized and multidisciplinary work-up which included history taking and cognitive examination by a neurologist, assessment of vital functions, informant-based history, and assessment of needs by a specialized dementia nurse, neuropsychological investigation, brain magnetic resonance imaging, electroencephalogram, standard laboratory work, and generally a lumbar puncture for CSF biomarker analysis. Some patients underwent amyloid PET scans instead of CSF collections. All patient cases were reviewed in a multidisciplinary meeting at which findings were reviewed toward arriving at a consensus on a diagnosis and treatment plan (18, 19). Diagnoses of Alzheimer’s dementia required an abnormal CSF biomarker profile or positive amyloid PET scan (21, 22). Amyloid PET scans utilized either [^18^F]Florbetaben or [^18^F]Florbetapir and were classified as amyloid positive based on the presence of fibrillary amyloid pathology in the neocortex as evaluated by visual rating by a nuclear medicine physician. CSF Alzheimer’s biomarkers were measured with Roche Elecsys P-Tau 181/Abeta42 assays (510k K221842) using a cut point of 0.02 for amyloid positivity (23) or with Fujirebio Innotest ELISAs p-Tau 181/Aβ42 using a cut point of 0.06 (24). Whole blood was obtained from each subject through vena puncture and processed into plasma by centrifuging at 1,800×*g* for 10 min at 20°C. Processed K2EDTA plasma samples were aliquoted in 0.5 mL-portions in polypropylene tubes and stored at −80°C in the biobank until dry-ice transportation to the Quanterix CLIA lab for Simoa p-Tau 217 testing. The intended use population of the test is objectively impaired individuals. Accordingly, cases diagnosed with MCI (*n* = 229) and AD (*n* = 123) were chosen to comprise a portion (40%) of the training and validation cohorts. Details of these subgroups have been previously reported (24) and are shown in aggregate in Supplementary Table S1. In addition, 50 each of cases diagnosed with frontal temporal dementia (FTD) and dementia with Lewy bodies (DLB) were examined. A proportion of these samples were also amyloid positive, and the accuracy of the test for detection of amyloid in these mixed pathology cases was characterized.

#### 2.6.2 Bio-Hermes cohort

From April 2021 through November 2022, 17 research sites prospectively recruited and enrolled consented study participants from their community-based populations. The clinical sites were recruiting centers for clinical trials investigating new drug treatments for Alzheimer’s. A key goal of the Bio-Hermes cohort was enrichment for ethnic/racial diversity. Participants who met inclusion criteria (20) were identified as belonging to one of the three clinical cohorts: cognitively unimpaired, MCI, and mild AD. Participants stratified to the MCI cohort met the following criteria: a diagnosis of MCI based on NIA-AA criteria (25) and verified through medical records, or had screening results as follows: MMSE score of 24 to 30 inclusive; RAVLT-delayed recall Score of at least 1 SD below the age-adjusted mean; and in the investigator’s judgment, minimal to mild functional impairment but with preservation of independence in functional abilities based on the FAQ score/study partner report. Participants stratified into the mild Alzheimer’s cohort met the following criteria: a diagnosis of probable Alzheimer’s based on the NIA-AA criteria (25) and verified through medical records, OR had screening results as follows: MMSE score of 20–24; RAVLT-delayed recall Score ≥1 SD below the age-adjusted mean; and in the investigator’s judgment, evidence of functional decline and dependence in functional abilities based on FAQ score/study partner report. Amyloid PET scans were obtained for all Bio-Hermes participants following clinical diagnosis at designated imaging facilities near the recruitment sites. PET scans were conducted at a designated imaging facility near each site using [^18^F]Florbetapir tracer. For consistency of PET scan interpretations, all scans were uploaded into an imaging portal accessible for visual reading by IXICO Technologies Inc. whereby the reader had visibility to a subject’s standardized uptake value ratio value but made the final determination according to manufacturer standards. Underrepresented population groups included Hispanic participants and non-Hispanic Black participants, overall constituted 27.8% of the symptomatic sub-cohort (MCI and mild Alzheimer’s). Whole blood samples were obtained from each subject through vena puncture in K2EDTA tubes, processed into plasma, and placed in the −80°C freezer within 4 h. Samples were shipped on dry-ice to the Quanterix CLIA lab for Simoa p-Tau 217 testing. To meet the intended use population, cases diagnosed with MCI (*n* = 286) and mild AD (*n* = 235) were chosen to comprise a portion (60%) of the training and validation cohorts. Details of these subgroups have been previously reported (20) and are shown in aggregate in Supplementary Table S1.

#### 2.6.3 Diagnostic threshold development and validation

To align with current recommendations for confirmatory plasma test performance (5, 7, 26), we endeavored to establish two cut points and achieve a minimum of 90% accuracy for the Simoa p-Tau 217 test on cohorts with objective cognitive symptoms. To do this, we utilized the samples from the MCI and AD groups within each cohort (ADC *n* = 352, Bio-Hermes *n* = 521) and randomized the samples of both cohorts combined into a training and validation sets stratified by MCI and AD status. The p-Tau 217 results from the validation were kept separate and blinded until use for validation. Diagnostic thresholds were modeled with the objective of achieving the accuracy target while minimizing the intermediate zone between the two cutoffs. The use of both cohorts for establishing the thresholds was deliberate to include the maximum diversity into the threshold setting. This diversity leads to the robustness of the thresholds in clinical practice.

#### 2.6.4 Plasma sample analysis

Prior to analysis, K2EDTA plasma samples were thawed at room temperature for 60 min and centrifuged at 10,000*g* for 10 min. Subsequently, p-Tau 217 concentrations from the clarified plasma supernatant were measured in duplicate on the Simoa HD-X analyzer in batches according to Quanterix CLIA laboratory SOPs using a single lot of reagents. All samples were tested in blinded fashion without knowledge of any clinical information, with unblinding occurring only after all Simoa testing was completed.

#### 2.6.5 Statistical methods

Analytical study analyses followed the statistical techniques recommended in the appropriate CLSI guideline. Reporting of clinical performance metrics follow standard statistical practice, including effect sizes with 95% confidence intervals for key measures. *p*-values reported for comparisons of means are based on t-tests. *p*-values for comparisons of categorical variables are from likelihood ratio test of homogeneity. Pairwise comparisons of means for discerning race/ethnicity group differences were performed by Tukey–Kramer test. Receiver operating characteristics-areas under the curve (AUCs) were calculated for comparisons of clinical performance.

To set clinical thresholds, a four factor, 40-run space filling design was used to model p-Tau 217 performance across false positive and false negative rates. The four design factors were the scale and shape parameters of log-normal distributions for amyloid negative and amyloid positive samples with the factor ranges determined by the 25th and 75th percentiles of 500 simulated distributions. A prediction profiler with a desirability function was used to evaluate and optimize the predicted performance (sensitivity, specificity % in indeterminant zone) in terms of false positive and false negative rates. The optimal rates were then converted to p-Tau 217 thresholds based on the fitted log-normal distributions. Statistical software used was JMP Pro 18.

## 3 Results

### 3.1 Analytical performance

#### 3.1.1 Does response and linearity

**Figure 1:**
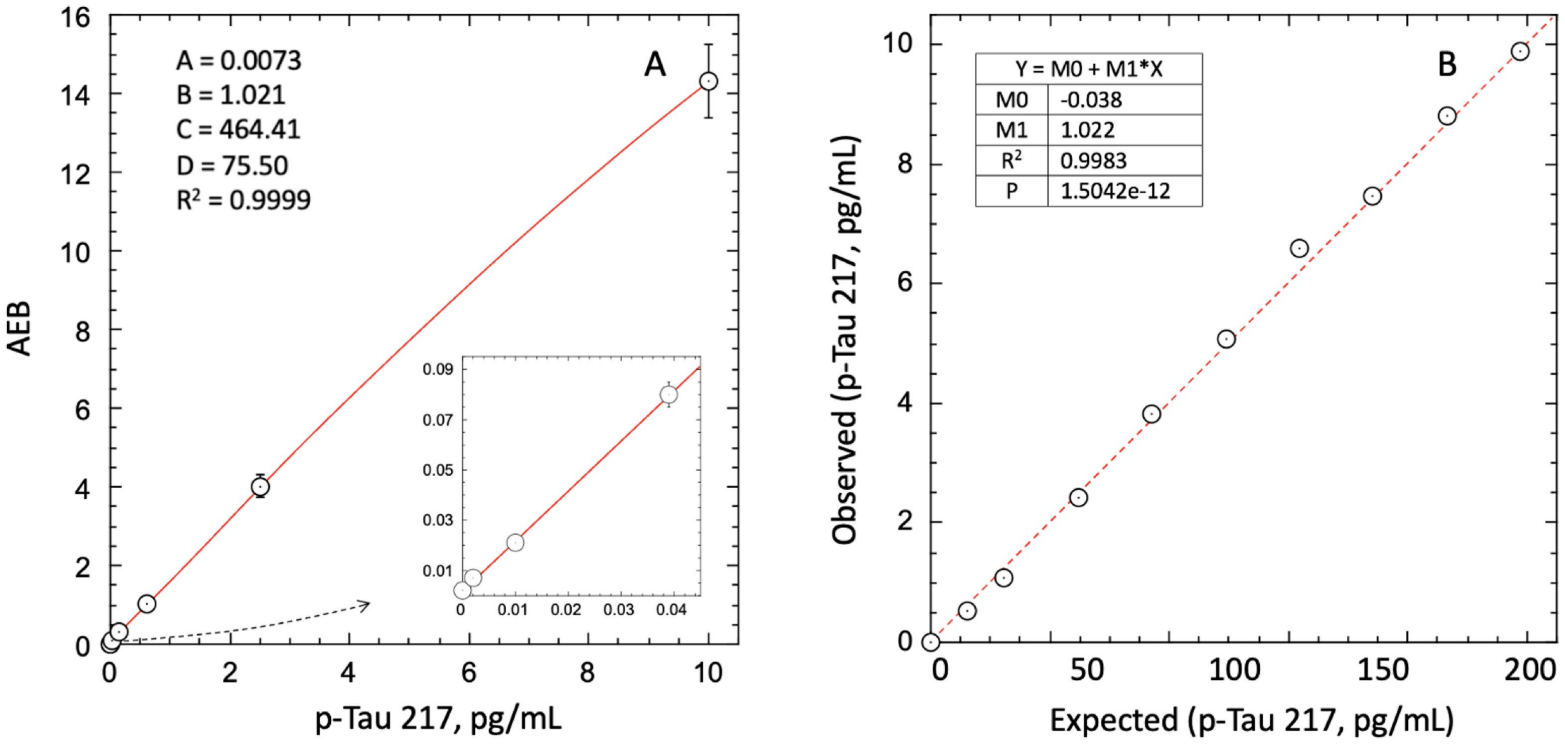
Dose response and linearity of Simoa LucentAD p-Tau 217 assay. **(A)** 4-parameter logistic fit of average enzymes/bead (AEB) signal from mean of duplicate calibrator replicates. **(B)** Linear regression analysis of linearity across high:low sample admixtures showed an average bias of 4%, with no significant improvement from a polynomial fit. Data represent mean of triplicates.

Figure 1 shows a representative calibration curve across a 3-log range. The low background typical for Simoa digital immunoassays is highlighted in Figure 1A. Linearity, evaluated across descending ratios from 1.0 high sample:low sample (ratio of 1.0 equals 100% AD (high) pool) to 0.875, 0.75, 0.625, 0.5, 0.375, 0.25, 0.125, 0.0625, and 0 (i.e., 100% CN pool), is depicted in Figure 1B. The average bias from expected values across the admixtures was 4%, with no improvement in fitting accuracy using a polynomial instead of linear fit. Linear regression statistics are depicted in Figure 1B. Native high p-Tau 217 samples diluted with sample diluent recovered within 80–120% of expected across serial dilutions to 16× dilution (not shown).

#### 3.1.2 Sensitivity

**Figure 2:**
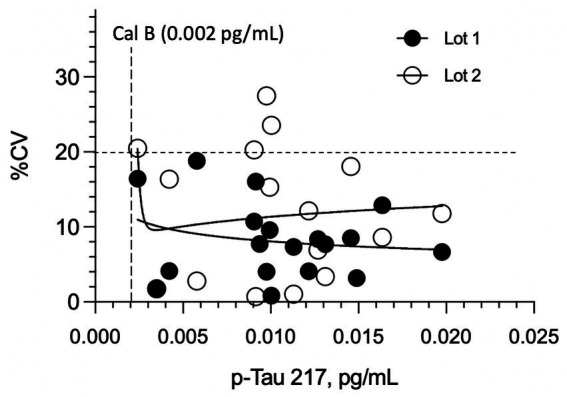
Imprecision (CV%) of plasma p-tau217 measurements in cognitively normal individuals across two reagent lots. LLoQ was established using 18 healthy donor plasma samples (near anticipated LLoQ) tested in duplicate over two runs with different reagent lots. No significant difference in overall imprecision was observed between Lot 1 and Lot 2 (*p* = 0.264). All replicate CVs for Lot 1 remained below 20%, even at concentrations <0.003 pg/mL. The estimated analytical LLoQ for Lot 2, determined by the power fit reaching the 20% imprecision threshold, was 0.003 pg/mL.

The highest LoB, LoD, and LLoQ results for the two reagent lots are reported for the assay. The highest LoB was determined to be 0.0005 pg/mL (0.5 fg/mL), and the highest LoD was calculated to be 0.0015 pg/mL. For LLoQ, precision profiles for repeated measurements of 18 native plasma samples from healthy donors are depicted in Figure 2. Most of the data exhibited less than 20% replicate CVs, hence with the reagent lot 1 data set and LLoQ could not be satisfactorily fit. Lot 2, however, gave a power fit that intersected the 20% CV threshold at 0.003 pg/mL. Correcting for a 1:2 pre-dilution of samples used in the instrument protocol, this yielded a functional LLoQ of 0.006 pg/mL. (Note: The 1:2 pre-dilution is not included in the definitions of LoB and LoD because these analytical estimates are not used within the reportable range for quantifying the analyte.) Accuracy of concentration readouts in this part of the assay range was verified by confirming readback of the lowest p-Tau 217 calibrator (0.002 pg/mL) was within 80–120% with CVs of 13–22%. Despite only one reagent lot having sufficient imprecision to define an LLoQ, the LLoQ determined using the worst-performing lot (Lot 2) is considered applicable to Lot 1.

#### 3.1.3 Repeatability and reproducibility

Repeatability and within-lab reproducibility for a panel of 6–8 amyloid negative and positive K2EDTA plasma samples spanning the lower and upper diagnostic cutoffs (as would be encountered in the intended use population) are summarized in Table 1. In both studies, percent coefficients of variation were ≤18%, even down to a level of 0.01 pg/mL, which is near the LLoQ and 4-fold lower than the lowest diagnostic cutoff (0.04 pg/mL, see Diagnostic Thresholds).

**Table 1:** Assay repeatability and reproducibility.

Sample	Mean (pg/mL)	SD (pg/mL)	%CV
Intra assay repeatability (1 lot, 2 instruments, 2 analysts)			
Plasma level 1	0.010	0.001	12.7
Plasma level 2	0.101	0.011	11.2
Plasma level 3	0.043	0.004	8.5
Plasma level 4	0.052	0.003	5.7
Plasma level 5	0.044	0.002	4.8
Plasma level 6	0.060	0.005	8.0
Inter assay reproducibility (1 lot, 2 instruments, 2 analysts)			
Plasma level 1	0.011	0.002	18.0
Plasma level 2	0.113	0.012	10.6
Plasma level 3	0.044	0.004	10.1
Plasma level 4	0.050	0.004	8.6
Plasma level 5	0.039	0.003	8.7
Plasma level 6	0.059	0.009	15.8
Plasma level 7	0.396	0.015	3.8
Plasma level 8	0.136	0.021	15.5

Precision (repeatability and within-laboratory) was evaluated using a 5-day × 2 run × 2 replicate design (40 replicates) on 5 native K2EDTA plasma samples (low, mid, high) across 2 instruments/analysts. Low/high contrived QC samples (CSF-spiked) were included. Intra-assay repeatability used 20 replicates each of 5 normal K2EDTA plasma samples, with average CV analysis. Abbreviations: SD, standard deviation; CV, coefficient of variance.

#### 3.1.4 Specificity

**Figure 3:**
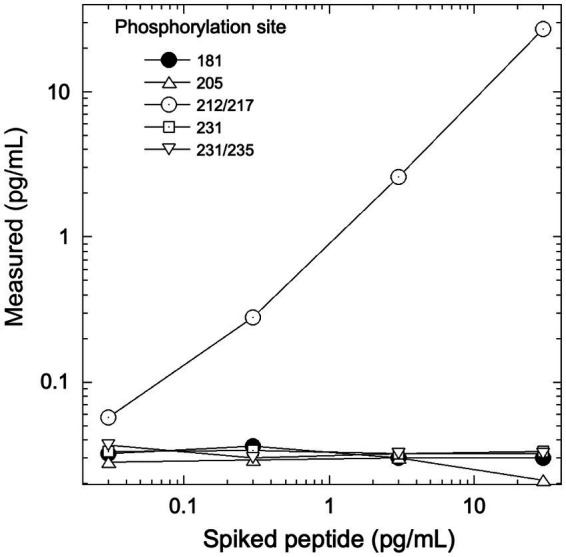
Assay response to tau peptides phosphorylated at different residues. Each peptide included an unphosphorylated N-terminal epitope along with 1 or 2 phosphorylated epitopes at the sites indicated. The assay was unreactive to sites other than 212/217. PT3 antibody reactivity to phosphorylation at the 212 vs. 217 sites was characterized previously (11).

Figure 3 depicts the assay response to peptides phosphorylated at different amino acid residues. All p-tau peptides other than the peptide phosphorylated at the 212 and 217 residues peptide yielded a mean cross reactivity of <5%. The positive control (212/217) gave a recovery of 88.2% of the expected concentration.

#### 3.1.5 Endogenous interferences

Physiologically relevant levels of 8 potentially interfering endogenous substances (triglycerides, hemoglobin, total protein, conjugate and unconjugated bilirubin, HAMA, rheumatoid factor, and biotin) were tested by spiking into 3 plasma samples with p-tau 217 concentrations spanning the lower and upper diagnostic cutoff’s (0.04, 0.09 pg/mL respectively) as would be encountered in the intended use population. Table 2 exhibits the observed percent differences between spiked and un-spiked control samples, with overall mean % differences across the samples between −2.3 to 6.6%, with minimum and maximum % differences ranging from −11.8 to 15.8%.

**Table 2:** Endogenous interferences.

Observed % difference between spiked and control specimen					
Potential interferent	Level (Spiked)	Low positive plasma (0.024 pg/mL)	Moderately Positive Plasma (0.060 pg/mL)	High positive Plasma (0.114 pg/mL)	Mean % Difference
Triglycerides	1,000 mg/dL	5.4	4.3	−7.4	0.8
Hemoglobin	500 mg/dL	6.2	6.9	6.6	6.6
Total protein	1 g/dL	−11.8	0.0	4.8	−2.3
Conjugated bilirubin	20 mg/dL	0.6	10.6	1.9	4.3
Unconjugated bilirubin	20 mg/dL	0.4	10.7	1.7	4.3
HAMA	10 ng/mL	−2.0	15.8	6.1	6.6
Rheumatoid factor	95 U/mL	6.6	9.2	−0.1	5.2
Biotin	0.360 mg/dL	−4.9	9.2	−4.4	0.0

Assessment of the impact of endogenous interferents (bilirubin, triglycerides, etc.) on three K2EDTA plasma samples (0.024, 0.060, 0.114 pg/mL) using one reagent lot and instrument. Interferent stock solutions (≥20× target concentration) were spiked into samples at a 1:19 ratio (interferent:sample, ≤5% matrix dilution). Solvent alone was added to controls. Total protein was directly added; HAMA was diluted into the matrix. All samples and controls were tested in duplicate within the same assay.

#### 3.1.6 Sample stability

p-Tau 217 as measured by the Simoa p-Tau 217 assay was found to be stable with a maximum average difference between test condition and control condition of 9% for 3 freeze–thaw cycles, 48 h of refrigerated storage, and 8 h of room temperature storage (Table 3).

**Table 3:** Stability of p-Tau 217 in EDTA plasma samples.

Difference between test and control condition (pg/mL)			
Condition	Average % Difference	Lower 95% CI	Upper 95% CI
F/T 1	4.6	−0.2	9.4
F/T 2	2.1	0.2	4.0
F/T 3	5.1	−0.9	11.1
2-8°C, 24 h	3.8	1.1	6.6
2-8°C, 48 h	3.7	−1.8	9.3
RT, 4 h	2.7	−0.8	6.2
RT, 8 h	8.9	3.4	14.4

Stability of 6 native K2EDTA plasma specimens (0.024–0.114 pg/mL) was assessed at room temperature (4, 8 h), refrigerated (2–8°C; 24, 48 h), and after 3 freeze–thaw cycles, using one reagent lot/instrument. −70°C storage served as the control.

### 3.2 Clinical performance

#### 3.2.1 Demographic and clinical characteristics

**Figure 4:**
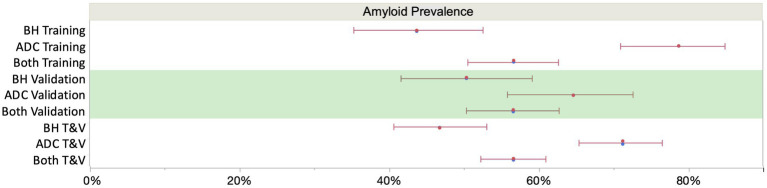
Sub cohort prevalences (with 95% score proportion CI’s). The overall mean prevalence was 56% across the two cohorts, which is skewed upward by the selected amyloid positives among the Amsterdam cohort. Assuming an intermediate prevalence of 50% (7), overall NPV and PPV were 90.4 and 91.4%, respectively, at the selected cutoffs (Figure 6). BH, Bio-Hermes; ADC, Amsterdam Dementia Cohort.

K2EDTA plasma samples from the ADC (*n* = 352) and study participants enrolled in the Bio-Hermes study (*n* = 521) were analyzed for p-Tau 217 and the results were compared with amyloid status by either CSF biomarkers or visual amyloid PET. The demographic and clinical characteristics of the two cohorts combined and separated by amyloid status are depicted in Table 4. Supplementary Table S1 shows the demographics split by the original two cohorts. In the combined cohort (all data), the mean age was 70.1 (SD 8.0) years, with 50.3% female representation. However, the ADC reflected a younger population with a mean age of 65.4 years (SD 7.7, range 43–83), while the mean age in the Bio-Hermes cohort was 73.2 (SD 6.6, range 59–85) (Supplementary Figure S1). Overall, most of the participants were white (86.6%), but 11.1% of study participants from Bio-Hermes were of black or African American origin. 13.1% of Bio-Hermes participants were Hispanic or Latino, with 27.8% of the participants in this cohort representing under-served minorities in total (including Asian participants, Pacific Islander participants and Native American participants) (20). All individuals were symptomatic following the inclusion criteria of the study, with a diagnosis of either MCI (59%) or AD (including probable AD) (41%). 47.3% had one or more copies of apoE4 (APOE carriership). Overall, 56.7% of the participants were positive by either amyloid PET or CSF biomarkers. A breakdown of amyloid prevalence by subgroup is depicted in Figure 4. The prevalences differed significantly between the two cohorts. In the ADC, 56.3% of MCI subjects and by design >99% of the dementia patients were amyloid positive. The MCI prevalence reflects all comers to the Amsterdam tertiary care clinic, and the high prevalence among dementia subjects is due to selection of this clinical subgroup in which the diagnosis was confirmed by CSF biomarker results. On the other hand, 35.0% of the MCI subgroup in Bio-Hermes was amyloid positive, while 61.3% of the dementia subgroup was positive (Supplementary Table S2). These comparatively lower numbers may reflect differing diagnostic criteria, use of recruited participants who had not been previously evaluated for cognitive symptoms, and clinical diagnoses being made prior to PET testing.

**Table 4:** Demographic characteristics.

	Aβ+	Aβ−	All	*p*-value
Age				0.1893
Mean (SD)	70.4 (7.9)	69.6 (8.2)	70.1 (8.0)	
Range	45–85	43–85	43–85	
Sex				0.1016
Female	261 (52.7%)	178 (47.1%)	439 (50.3%)	
Race				0.0037
American Indian or Alaska Native	0 (0.0%)	1 (0.3%)	1 (0.1%)	
Asian	5 (1.3%)	5 (1.7%)	10 (1.5%)	
Black or African American	18 (4.8%)	42 (14.0%)	60 (8.9%)	
Native Hawaiian or other Pacific Islander	0 (0.0%)	1 (0.3%)	1 (0.1%)	
Other/Unknown	10 (2.7%)	9 (3.0%)	19 (2.8%)	
White	343 (91.2%)	243 (80.7%)	586 (86.6%)	
Missing^†^	119	77	196	
Ethnicity				0.0890
Hispanic or Latino	33 (13.52%)	35 (12.64%)	68 (13.05%)	
Not Hispanic or Latino	205 (84.02%)	241 (87.00%)	446 (85.60%)	
Not reported	6 (2.46%)	1 (0.36%)	7 (1.34%)	
Unknown (ADC)	251	101	352	
Diagnosis				<0.0001
AD	122 (24.5%)	1 (0.3%)	123 (14.1%)	
Probable AD	147 (29.5%)	93 (24.5%)	235 (26.9%)	
MCI	229 (46.0%)	286 (75.3%)	515 (59.0%)	
MMSE				<0.0001
Mean (SD)	24.1 (4.3)	26.2 (2.7)	25.0 (3.8)	
Range	2–30	16–30	2–30	
Missing data	2	2	4	
APOE e4 carrier/non carrier				<0.0001
E2E2	0 (0.0%)	2 (0.5%)	2 (0.2%)	
E2E3	11 (2.2%)	53 (13.9%)	64 (7.3%)	
E2E4	10 (2.0%)	7 (1.8%)	17 (1.9%)	
E3E3	141 (28.3%)	239 (62.9%)	378 (43.3%)	
E3E4	236 (47.4%)	65 (17.1%)	298 (34.1%)	
E4E4	92 (18.5%)	6 (1.6%)	98 (11.2%)	
Missing	8 (1.6%)	8 (2.1%)	16 (1.8%)	
Plasma p-tau217 Concentration (pg/mL)				<0.0001
Median (SD)	0.132 (0.09)	0.046 (0.04)	0.09 (0.08)	
Range	0.006–0.776	0.006–0.549	0.006–0.776	
Amyloid status source				<0.0001
CSF	242 (48.6%)	95 (25.0%)	337 (38.6%)	
PET	253 (51.1%)	283 (74.9%)	536 (61.4%)	

Demographics of combined cohort. Amyloid classification based on amyloid PET or CSF biomarkers. P-values are all 2-sided with no adjustment for multitude of tests. Aβ, amyloid beta; MCI, mild cognitive impairment; MMSE, Mini-Mental State Examination; ADC, Amsterdam Dementia Cohort; SD, standard deviation; PET, positron emission tomography.

† Ethnicity data from 196 participants in the Amsterdam Dementia Cohort were unavailable.

#### 3.2.2 p-Tau 217 measurement in plasma samples

**Figure 5:**
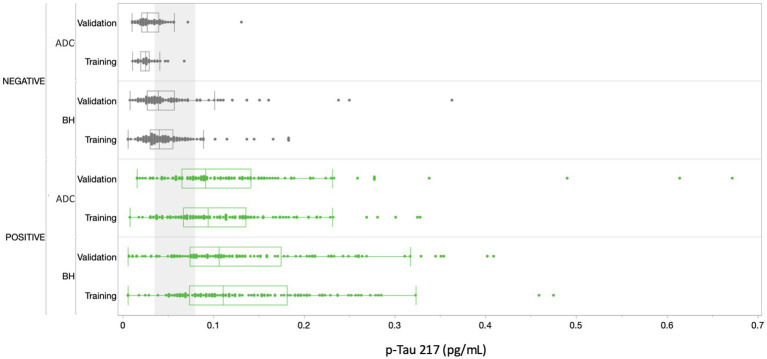
Distribution of results across all cohorts. Amyloid positives are depicted in green, amyloid negatives in gray. The gray shading corresponds to the ~30% intermediate zone of uncertainty between lower and upper cutoffs of 0.04 and 0.09 pg/mL, respectively (Figure 6). BH, Bio-Hermes; ADC, Amsterdam Dementia Cohort.

Figure 5 depicts p-Tau 217 sample results broken out by cohorts and subgroups. 100% of the samples were above the assay LoD and gave a reportable result. 99.5% of the samples were above the assay LLoQ and were thus quantifiable with acceptable precision. The median concentration of plasma p-Tau 217 was 2.87-fold higher in amyloid-positive study participants (amyloid negative 0.046 pg/mL, SD 0.04; amyloid positive 0.132 pg/mL, SD 0.09, *p* < 0.0001) and the differentiation between amyloid-positive and amyloid-negative study participants gave an overall AUC of 0.89 (0.87–0.92). There was a notable difference in discrimination between the ADC and Bio-Hermes cohorts, with the ADC training and validation subgroups yielding AUCs of 0.96 (0.94–0.99) and 0.93 (0.89–0.96) respectively vs. Bio-Hermes training and validation subgroups yielding AUCs of 0.89 (0.85–0.93) and 0.84 (0.78–0.89) respectively. There may be multiple reasons why the observed performance was slightly different between the two cohorts. One potential reason is the greater racial/ethnic diversity of the Bio-Hermes cohort may have negatively impacted the diagnostic accuracy, although the racial/ethnic subgroup analysis did not reveal statistically significant differences (next section). Another potential explanation may be the presence of a larger number of comorbidities influencing the results in the older Bio-Hermes population. Detailed comorbidity information for the Bio-Hermes cohort was not available. It is also noted that Bio-Hermes utilized visual amyloid PET as the reference method, while most of the Amsterdam cohort utilized CSF biomarkers. It is unclear if visual amyloid PET may have introduced greater uncertainty in amyloid status than quantitative CSF classification. Yet another potential reason for the observed differences may be the underlying methods by which the cohort individual subjects were assessed for clinical status. A breakdown of performance metrics for various combinations of data sets is given in Supplementary Table S3.

In addition to the training and validation cohorts, subgroups of 50 each of diagnosed FTD and DLB cases were tested. Demographic and clinical details of these cases can be found in Supplementary Table G1. A proportion of these cases exhibited amyloid positivity (22% for FTD, 50% for DLB). Despite the limited statistical powering from the small sampling sizes, the data suggest amyloid detection accuracy statistically consistent with the validation cohort for detecting amyloid in DLB and FTD cases (85.0 and 87.5% respectively, Supplementary Table G4). In addition, inclusion of all 100 cases to the validation cohort had no statistically significant effect on the performance of the test in classifying amyloid status (Supplementary Figure G1).

#### 3.2.3 Clinical thresholds

**Figure 6:**
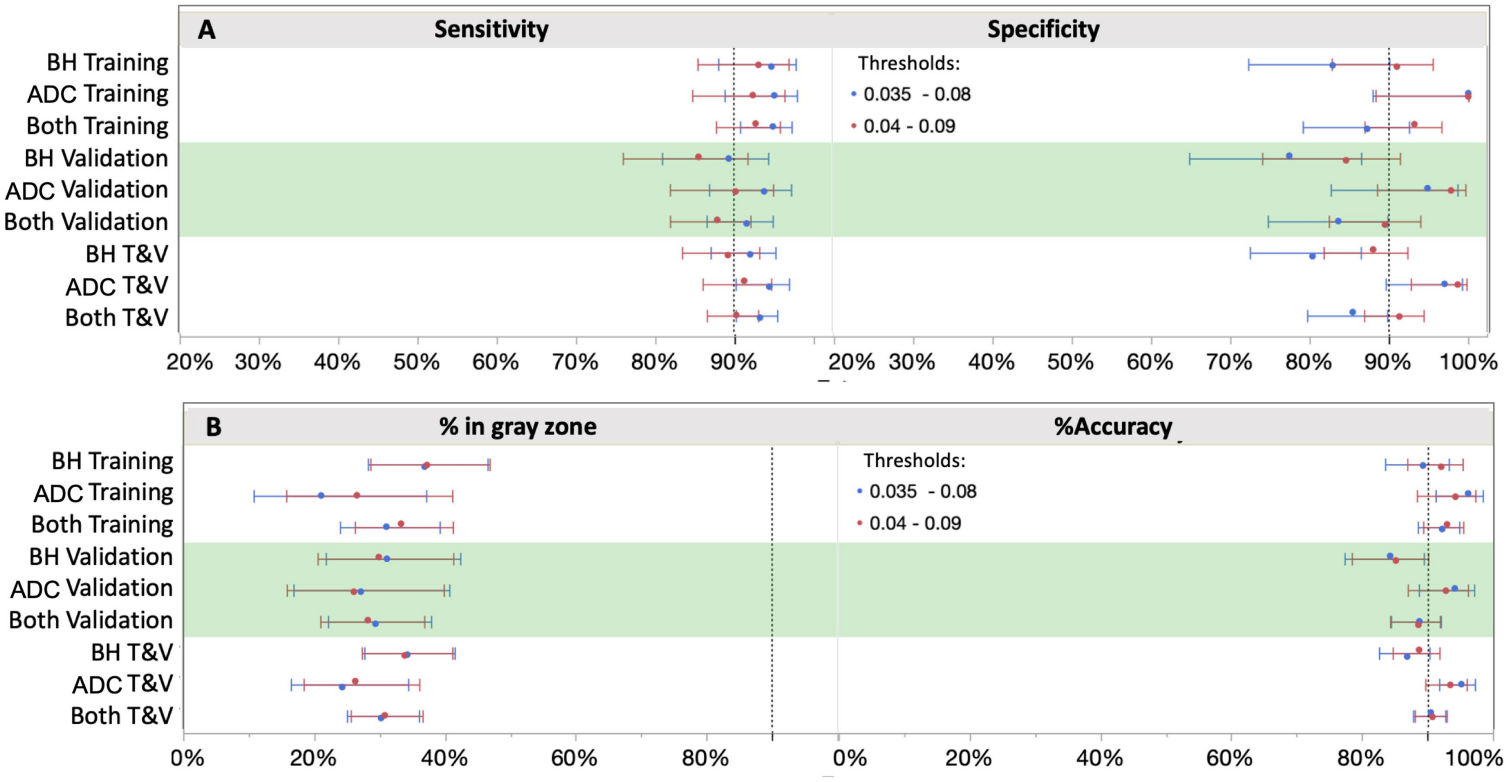
Clinical performance with two different cut-off scenarios. Shifting the cutoffs downward (blue) increased sensitivity, but at a higher cost to specificity. 0.04 and 0.09 pg/mL provided the best balance for both ruling out and ruling in with high confidence (≥90%), thus capturing both ends of the disease spectrum. BH, Bio-Hermes; ADC, Amsterdam Dementia Cohort.

In setting the lower and upper diagnostic thresholds, the objective was to maximize assay accuracy while minimizing the intermediate zone with an intended use population of objectively symptomatic individuals (MCI and AD). A simulation study was used to optimize the setting of the thresholds. Two threshold pairs representing the best balance were identified. Figure 6 depicts the clinical performance of the 0.035/0.080 pg/mL threshold pair, and a slightly higher 0.040/0.090 pg/mL threshold pair with respect to sensitivity, specificity, accuracy, and intermediate ranges across the subgroups. Note: sensitivity and specificity are reported here when excluding samples in the intermediate zone. In general, the lower candidate threshold pair favored sensitivity, while the higher threshold pair favored specificity (Figure 6A). Both candidate pairs gave similar performance for % intermediate zone and accuracy across the training subgroups. Generally, the wider spread of data observed in the Bio-Hermes cohort (Figure 5) contributed to a larger intermediate zone (~36%) than with the ADC (~25%). Combining cohorts gave an overall indeterminant range of ~30% irrespective of the choice of threshold pairs. Overall, the higher threshold pair (0.04, 0.09 pg/mL) struck the best balance, yielding sensitivity, specificity, and accuracy >90% across the full data set, as well as PPV and NPV > 90% with an amyloid prevalence of 50% representative of older patients with more concerning symptoms (7). As reflected in Table 5, the validation subgroups had reduced estimated clinical performance relative to the training subgroups, in particular the Bio-Hermes validation subgroup. The main driver behind the difference was a higher number of false negatives among the Bio-Hermes validation subgroup (16) vs. the training subgroup (5). A deeper look revealed no obvious non-random demographic factors among the split, and the difference appeared to be a matter of chance. We summarized the performance of the p-Tau 217 assay across both training and validation cohorts, as shown in Table 5. With the inclusion of all 873 patients across these two distinctly different independent cohorts, the test exhibited an overall accuracy of 90.7% excluding the intermediate range. The 30.9% intermediate range is mainly driven by the distribution spread introduced by the older, more diverse Bio-Hermes cohort. PPV and NPV depend on the prevalence of amyloid positivity in the population being tested. Supplementary Table S4 lists calculated PPV and NPV expected from populations with different disease prevalences, including the observed prevalence in this validation study (56%). In a population with low prevalence rates, such as among cognitively normal individuals or those with subjective cognitive decline (not yet validated), the Simoa p-Tau 217 test would exhibit a very high NPV (96–97%). Among patients with dementia where there is high prevalence of amyloid pathology, the test would exhibit a very high PPV (95%).

**Table 5:** Clinical performance of Simoa p-Tau 217 assay.

Performance category	Amsterdam training *n* = 165	Amsterdam validation *n* = 187	Bio-Hermes training *n* = 281	Bio-Hermes validation *n* = 240	ADC + Bio-Hermes *n* = 873
AUC	0.96 (0.94–0.99)	0.93 (0.89–0.96)	0.89 (0.85–0.93)	0.84 (0.78–0.89)	0.89 (0.87–0.92)
Sensitivity^†^	92.4% (86.6–93.1%)	90.2% (86.6–93.1%)	93.1% (86.6–93.1%)	85.6% (86.6–93.1%)	90.3% (86.6–93.1%)
Specificity^†^	100% (86.9–94.4%)	97.8% (86.9–94.4%)	91.0% (86.9–94.4%)	84.6% (86.9–94.4%)	91.3% (86.9–94.4%)
Accuracy^†^ (AA)	94.2% (88.0–92.9%)	92.8% (88.0–92.9%)	92.0% (88.0–92.9%)	85.1% (88.0–92.9%)	90.7% (88.0–92.9%)
Interm. range	26.7% (25.7–36.7%)	26.2% (25.7–36.7%)	37.4% (25.7–36.7%)	30.0% (25.7–36.7%)	30.9% (25.7–36.7%)

Clinical performance metrics with 95% confidence intervals of Simoa p-Tau 217 assay across two independent cohorts. AUC, area under the receiver operating characteristics curve; AA, Alzheimer’s Association.

† Excluding samples in the intermediate range.

#### 3.2.4 Race and ethnicity analyses

**Figure 7:**
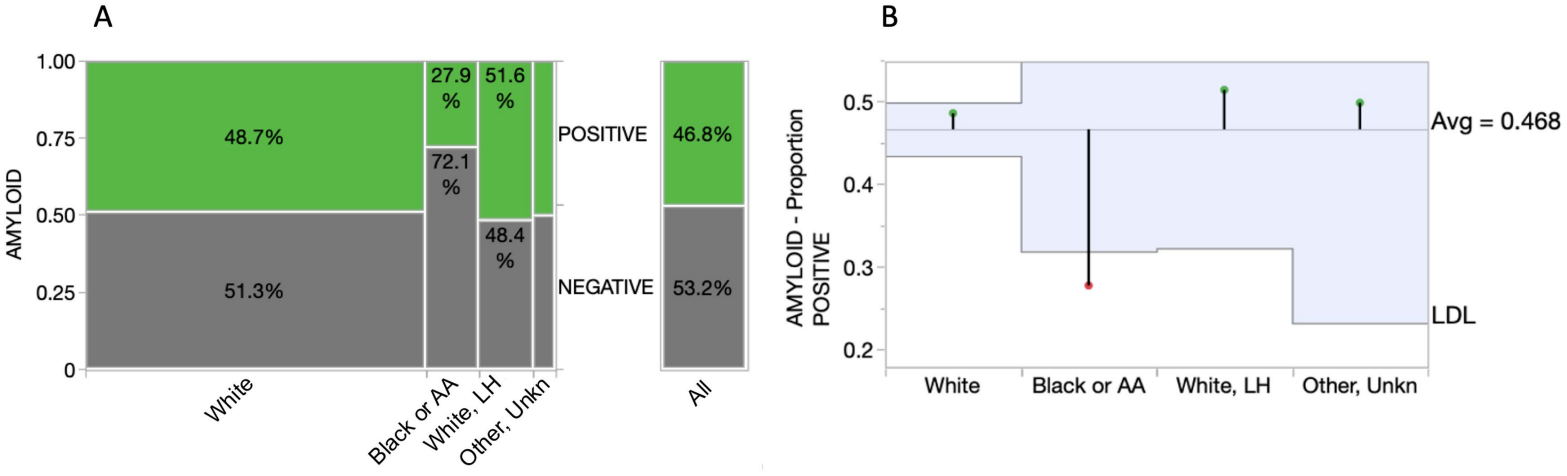
Comparison of amyloid positivity across racial/ethnic groups. **(A)** Mosaic plot illustrating a significantly reduced percentage of amyloid positivity among Black/AA participants. **(B)** Analysis of means for proportions graph highlighting statistical significance of the lower proportion of amyloid positivity among Black/AA participants (*p* = 0.0151; box boundaries reflect 95% CIs). AA, African American; LH, Latino/Hispanic; LDL, lower decision limit.

**Figure 8:**
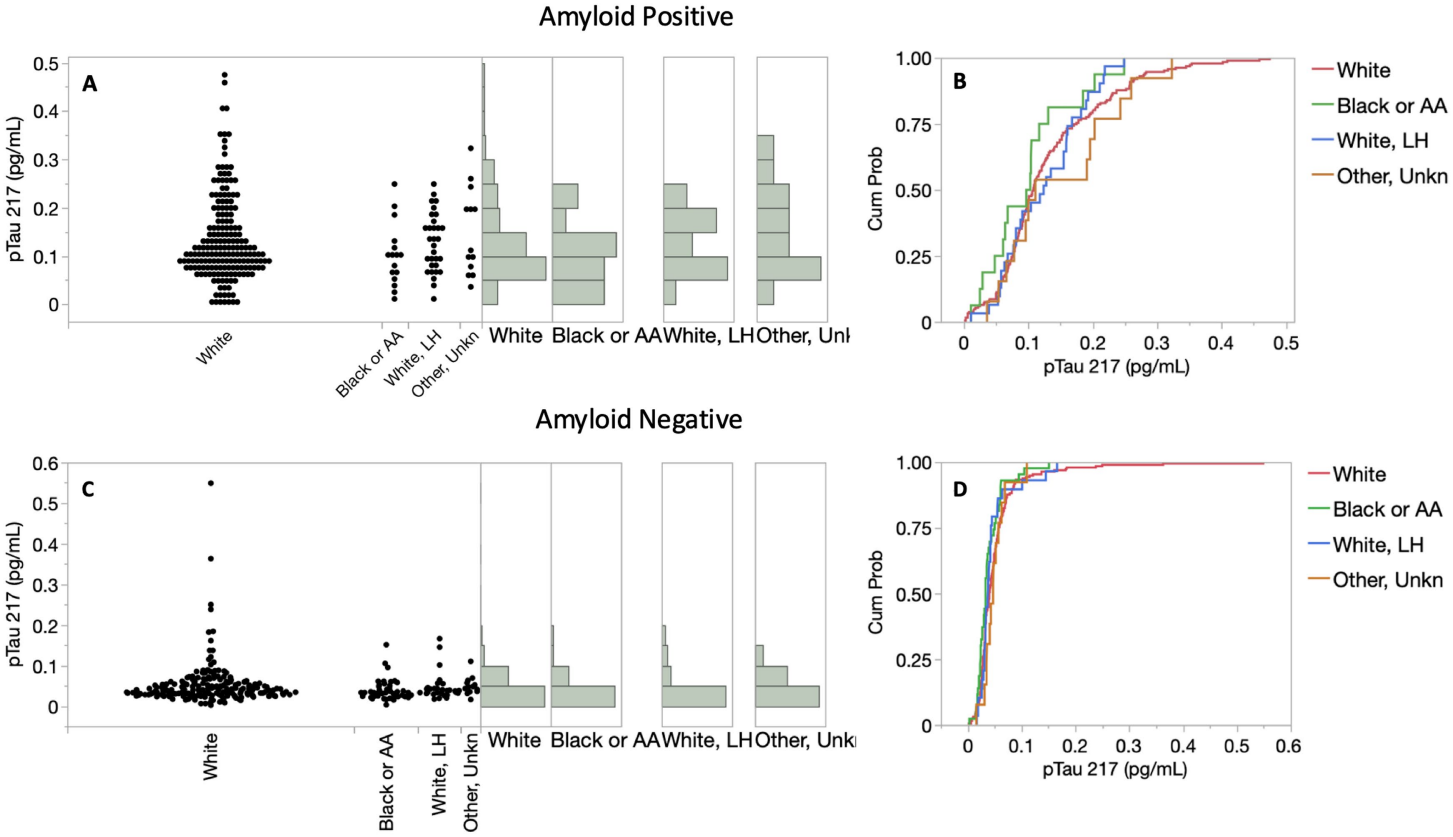
Distributions of p-Tau 217 results among racial/ethnic (R/E) groups in the symptomatic Bio-Hermes cohort. **(A,C)** p-Tau 217 distributions. p-Tau 217 did not differ significantly across R/E groups, as reflected by cumulative probability distribution functions depicted in panels **(B,D)**.

While the ADC was primarily white/European participants, Bio-Hermes represents a greater proportion of underserved racial/ethnicity (R/E) groups in the study cohort. The breakdown in R/E categories across all Bio-Hermes participants was approximately 74% white participants, 11% black/African American participants, 10% white/Latino participants, and 5% other/unknown. We attempted to discern whether there were any significant differences in test performance by examining p-Tau 217 levels for each R/E group in separate analyses. First, the proportions of clinical categories (MCI vs. AD) were not significantly different among the R/E groups (*p* = 0.0682). However, the amyloid positivity rate in the black/AA participant group was statistically lower as compared with other R/E groups (27.9% vs. ~50%, Figure 7A). The likelihood-ratio test *p*-value was 0.0151 and an analysis of means of proportions showed that the black/AA participant group had a lower rate of positivity as compared to the overall rate of 46.8% across the study (Figure 7B). Importantly however, p-Tau 217 results did not differ significantly across R/E groups (Figure 8). Comparing all R/E pairs using a Tukey–Kramer multiple comparison indicated that the difference in p-Tau 217 results were among the largest between white and black/AA participant groups, but these differences did not reach statistical significance for either the amyloid positive subjects (mean difference 0.033 pg/mL, *p* = 0.3827) or the amyloid negative subjects (mean difference 0.013 pg/mL, *p* = 0.1137) (Supplementary Table S5). Likewise, areas under the ROC curves ranged from 0.81 (0.67–0.96, black/AA participants) to 0.89 (0.76–0.96, white Latino participants) (Supplementary Figure S2). Exclusion of the black/AA participants did not significantly change the overall clinical performance. Given the limited powering of the R/E subgroups, additional powering and subgroup specific validation should be further explored.

## 4 Discussion

This report details the analytical and clinical validation of a simple, fully automated, and scalable digital immunoassay for accurate high sensitivity measurement of plasma p-Tau 217 that is suitable for routine clinical use. The test design and performance characteristics are aligned with the latest recommendations from expert groups on plasma test design and clinical performance capabilities needed to support confirmatory diagnostic use for identification of amyloid pathology in individuals with cognitive symptoms being evaluated for AD. In particular, the use of two rather than one diagnostic threshold has been recommended for plasma p-Tau 217 (5, 7, 26), and the feasibility and diagnostic performance of 2-threshold plasma p-Tau 217 tests in clinical practice scenarios have been shown (26, 27). Consistent with the findings presented here, the robust diagnostic performance of plasma p-Tau 217, often comparable to CSF and PET, has been further supported by a growing number of recent studies (26, 28–30). Critically, these same expert sources unanimously reflect a consensus that a diagnostic accuracy of ≥90% (defined as the sum of correct results per comparator divided by all results (5)) is considered functionally equivalent to FDA-cleared CSF biomarker tests and suitable to enable a diagnostic use-case for a plasma AD biomarker. As shown in this report, the Simoa p-Tau 217 test achieves this high-performance standard across a well-powered clinical study diverse in participant demographics, geographies, comparator methods, clinical settings, and race/ethnicities. The high level of performance extends to clinical sensitivity and specificity (90.3, 91.3%, respectively), which is comparable to amyloid PET. For comparison, against gold standard postmortem neuropathology, qualitative amyloid PET has achieved reported sensitivities and specificities of 88–98% and 80–95%, respectively (31, 32).

A notable difference in discrimination was observed between the ADC and Bio-Hermes cohorts, with the ADC training and validation subgroups yielding higher AUC and clinical performance parameters compared to Bio-Hermes. Multiple factors may contribute to this. One potential explanation is that the greater racial/ethnic diversity of the Bio-Hermes cohort might have influenced diagnostic accuracy, although the racial/ethnic subgroup analysis did not reveal statistically significant differences. Another factor could be the presence of a larger number of comorbidities in the older Bio-Hermes population, for which detailed information was unavailable. The use of visual amyloid PET as the reference method in Bio-Hermes, versus predominantly quantitative CSF biomarkers in the ADC, may also have introduced greater uncertainty in amyloid status. Additionally, differences in the methods used to assess clinical status across the cohorts could have played a role. As reflected in Table 5, even within the same sample cohorts, differences between training and validation subgroups suggest that with sample sizes in the 240–280 range, significant variations in clinical performance parameter estimates can emerge due to chance, highlighting the importance of adequately powered validation studies.

The clinical validation reported here builds upon several years of previously published data establishing the clinical validity of plasma p-Tau 217 for detecting amyloid and tau pathology using this assay, which was among the first immunoassay-based tests for plasma p-Tau 217. Prior studies have demonstrated its high accuracy compared to amyloid and tau PET (3) and CSF biomarker status (28), its ability to detect p-Tau 217 elevation early in the AD process (3), and its superior performance over p-Tau 181 and p-Tau 231 (28, 33). Illustrating the enhanced diagnostic utility of p-Tau 217, Therriault et al. (28) reported equivalent diagnostic performance for plasma and CSF p-Tau 217, whereas plasma p-Tau 181 (AUC 0.84) and p-Tau 231 (AUC 0.80) showed significantly lower performance compared to plasma p-Tau 217 (AUC 0.97). The Simoa p-Tau 217 assay was also shown to predict longitudinal cognitive changes as well as or better than amyloid or tau PET (3, 4), supporting its potential as a substitute for PET in clinical trial enrollment. In real-world clinical research, the assay achieved a high AUC against the CSF Aβ42/p-Tau ratio test (29). While this validation focused on symptomatic individuals, prior research indicates the assay’s accuracy in discriminating amyloid status in cognitively unimpaired older adults (3, 29), suggesting its potential utility across the entire AD continuum with further validation.

A key strength of this clinical validation study was the inclusion of two diverse independent cohorts, designed to represent a broad range of real-world variables, including geography, clinical settings, comparator methods, diagnostic criteria, amyloid positivity prevalence, and racial/ethnic diversity (Bio-Hermes). This heterogeneity likely makes the observed diagnostic performance parameters more reflective of real-world clinical practice. The strong performance in the ADC cohort (accuracy 93–94%, AUC 0.93–0.96) aligns with prior findings from a specialized center (29), potentially due to a more homogeneous population and stringent diagnostic criteria. The comparatively lower performance in the Bio-Hermes MCI cases might be attributed to the cohort’s diversity, inconsistent diagnostic criteria, potential for pre-PET misdiagnosis, higher comorbidity burden, and limitations of qualitative PET.

Supporting the assay’s inherent capabilities, its performance was statistically indistinguishable from an alternative Simoa p-Tau 217 assay (34) employing different antibodies. Yet a second report on the alternative assay suggested that a 20% intermediate zone was suitable for the cohorts tested (30). The broader intermediate zone of 30.9% obtained here, compared to the ~20–25% reported for other p-Tau 217 immunoassays (30), might reflect the real-world heterogeneity of the cohort. The presence of an intermediate zone in a diagnostic test can complicate clinical decision-making, as individuals falling within this range may require further investigations to definitively establish amyloid pathology status. Nevertheless, even with an intermediate zone, a plasma p-Tau 217 test offers a significant advancement by potentially substantially reducing the number of patients requiring more costly or invasive procedures. Recent data suggest that the ratio of plasma p-Tau 217/plasma Aβ42 could reduce the intermediate zone as compared with p-Tau 217 alone (35). However, a recent report has cautioned that the ratio of plasma p-Tau 217/Aβ42 as an assay readout carries significant pre-analytical risks, as Aβ peptides are labile, whereas p-Tau is relatively stable (30). On the other hand, the ratio of Aβ42/Aβ40 remains relatively unaffected, as both numerator and denominator are similarly affected by sample handling variables. To address the need for improved amyloid classification of intermediate zone cases with a more robust approach, a recent report of a multi-analyte algorithmic test that incorporates both p-Tau 217 and the Aβ42/Aβ40 ratio (along with two other AD-relevant plasma biomarkers) demonstrates that the intermediate zone in diverse populations can be significantly reduced by as much as 3-fold through the inclusion of additional biomarkers (36). The cohorts tested in the present study may therefore provide a realistic assessment of plasma p-Tau 217 clinical performance in a natural population.

p-Tau 217 is a low-abundance protein, posing analytical challenges in early AD stages. The high sensitivity of the digital Simoa assay used here overcomes this limitation, enabling reliable measurement across the AD spectrum, unlike some other methods with p-Tau 217 undetectable rates of 13–27%, including mass spectrometry and chemiluminescence methods (37, 38). Additionally, very low abundance p-Tau 217 is expected to be common in individuals with low amyloid burden, limiting the potential for tracking the biomarker longitudinally, for example in asymptomatic individuals such as those with SCD. Finally, methods with inadequate analytical sensitivity may not be suitable for precise assessment of biomarker status at or near a lower diagnostic threshold as would be needed for high confidence in ruling out the presence of amyloid pathology. Such tests may be limited to identifying only patients with high amyloid burden and sufficiently high plasma p-Tau 217 to support use as a rule-in test, potentially decreasing the benefit of a plasma biomarker test to reduce the number of more invasive tests and streamlining referrals.

The Bio-Hermes study’s goal of including at least 20% underserved populations revealed a lower amyloid positivity rate by PET in non-Hispanic Blacks, consistent with prior findings (20). It remains unclear why this has been observed, but perhaps it is related to differences in education levels and cognitive scoring that were found to be significant (20) combined with a tendency to over-diagnose in the absence of PET results. Also consistent was our finding among symptomatic individuals that differences in plasma p-Tau 217 between R/E groups did not attain statistical significance. As previously reported, p-Tau 181 and amyloid-beta ratio also did not differ between R/E groups (20). Importantly, the ideal plasma p-Tau 217 cutoffs for identifying amyloid status did not significantly differ between R/E groups in this study. It seems likely that discerning R/E differences requires larger and/or more diverse cohorts with greater power than the Bio-Hermes cohort provides. Nonetheless, it is reassuring that R/E differences in plasma p-Tau 217 seem to be absent to fairly minor. Differences in diagnostic performance based on sex, age, and apoE4 carriership were also found not to be significant (not shown). However, it is acknowledged that even small differences could have a significant impact when used for large-scale screening of populations. As the impact of demographic variables is explored more fully, guidance could be developed regarding the interpretation of p-Tau 217 test results in the context of these variables. Taken together the data here suggest that the results of the Simoa p-Tau 217 test can be similarly interpreted across different ethnicities, ages, sexes, and apoE4 genotypes.

The study is not without limitations. While the ADC represents tertiary care clinical practice and reflects all comers to the clinic without exclusions, the R/E composition was more limited to primarily to individuals of white European descent. On the other hand, the Bio-Hermes cohort was aimed at greater diversity, but the participants were recruited and evaluated at clinical research entities in a similar manner to therapeutic trial enrollment rather than at primary or secondary clinics. The Bio-Hermes study enrollment included various exclusions, including prior history of cancer, psychiatric conditions, recent alcohol dependence, other non-AD factors that could contribute to cognitive symptoms (e.g., bladder infection), underweight, potential competing neurological disorders, etc. In the Bio-Hermes cohort, comorbidities such as renal function, cardiovascular disease, and brain trauma were not captured or controlled for, potentially reducing the generalizability of the results. While the potential for co-morbidities to affect plasma biomarker concentrations has been a topic of considerable discussion in the context of clinical implementation of blood tests for AD, recent data suggest that the effect of what is generally considered the most impactful comorbidity—chronic kidney disease—may not be clinically meaningful for correct classification of amyloid status using plasma p-Tau 217 (39). Nonetheless, the potential for a higher prevalence of undocumented co-morbidities in the Bio-Hermes cohort composed of older participants and the emphasis on underserved minority participants may have been a contributing factor to the weaker diagnostic performance with this cohort relative to the ADC. Additional studies are ongoing to examine the effect of comorbidities on the Simoa p-Tau 217 test.

## 5 Conclusion

The Simoa p-Tau 217 blood test was clinically validated across two diverse independent cohorts of individuals with cognitive impairment. The test employs a two-cutoff design aligning with recently recommended high performance criteria for diagnostic confirmatory use, with an overall accuracy vs. amyloid PET and CSF of >90%, and sensitivity and specificity >90%. This two-cutoff design, with the cohorts studied here, led to an intermediate zone of ~30%. At an amyloid prevalence of 50%, reflecting mild cognitive impairment, the test also exhibited PPV and NPV greater than 90%. The test was analytically validated and shown to deliver single femtogram/mL sensitivity, enabling the measurement of plasma p-Tau 217 in all individuals tested. These results demonstrate that this Simoa plasma p-Tau 217 test as validated under CLIA is suitable for clinical use.

## Data Availability

The raw data supporting the conclusions of this article will be made available by the authors without undue reservation.
