# Supplementary material for: Analytical and clinical validation of a high accuracy fully automated digital immunoassay for plasma phospho-Tau 217 for clinical use in detecting amyloid pathology
Source: Front Neurol. 2025 Jul 9;16:1568971. doi: 10.3389/fneur.2025.1568971 (PMC12287614; doi:10.3389/fneur.2025.1568971)
Supplement: Supplementary file 1 [file Table_1.docx]

Analytical and Clinical Validation of a High Accuracy Fully Automated Digital Immunoassay for Plasma Phospho-Tau 217 for Clinical Use in Detecting Amyloid Pathology

**SUPPLEMENTAL MATERIALS**

**Table of Contents**

.

1. Figure S1: Age Distributions of Bio-Hermes and Amsterdam Dementia Cohorts……….…. 2
2. Figure S2: ROC Curves Across Racial/Ethnic Groups……………………………………… 3
3. Table S1: Demographic Characteristics by Cohort……………………………………….…. 4
4. Table S2: Amyloid Prevalence by Diagnostic Category……………………………….……. 5
5. Table S3: Performance Metrics for Various Combinations of Data Sets …………………… 6
6. Table S4: PPV and NPV of Simoa p-Tau 217 Assay by Prevalence ……………….………. 7
7. Supplemental Analysis: Accuracy of Simoa p-Tau 217 Assay for Dementia with Lewy

Bodies (DLB) and Frontal Temporal Dementia (FTD) Cases………...……………….……. 8

Table G1: Demographic and clinical characteristics of DLB and FTD………….…….. 8

Table G2: 2 x 3 Table for DLB cases……………………………………….…………. 8

Table G3: 2 x 3 Table for DLB cases…………………………………………….……. 8

Table G4: Performance metrics with 95% CI for DLB and FTD cases…………….…. 9

Table G5: Percentage of DLB and FTD cases included in validation cohort………….. 9

Figure G1: Effect of DLB and FTD samples on performance metrics………………… 10

Supplemental References………………………………………………………………. 10

1. Table S5: Tukey-Kramer Comparisons of Pairwise Differences in Mean p-Tau 217

Values Across Racial/Ethnic Groups………………………………………………………… 11


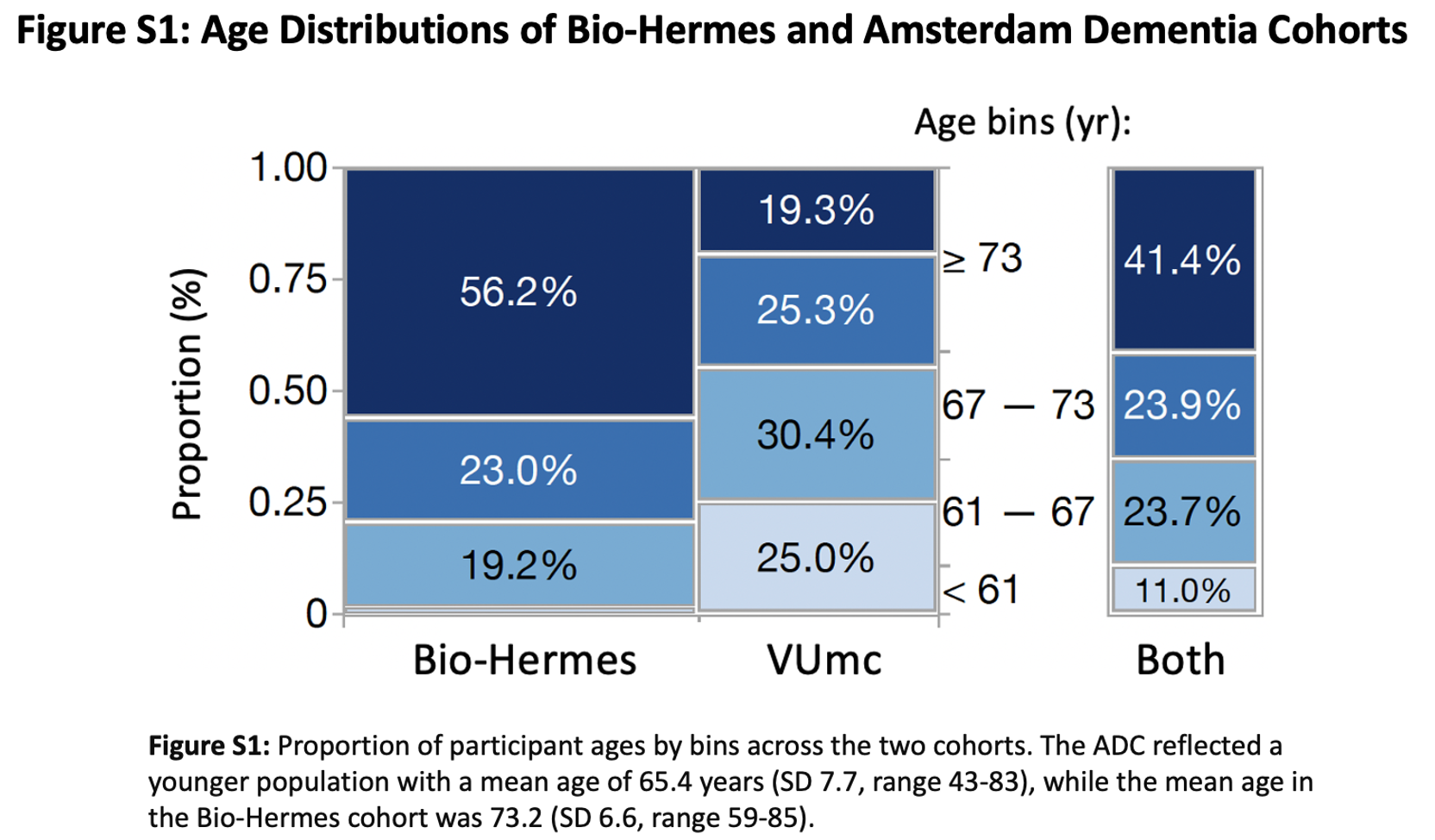


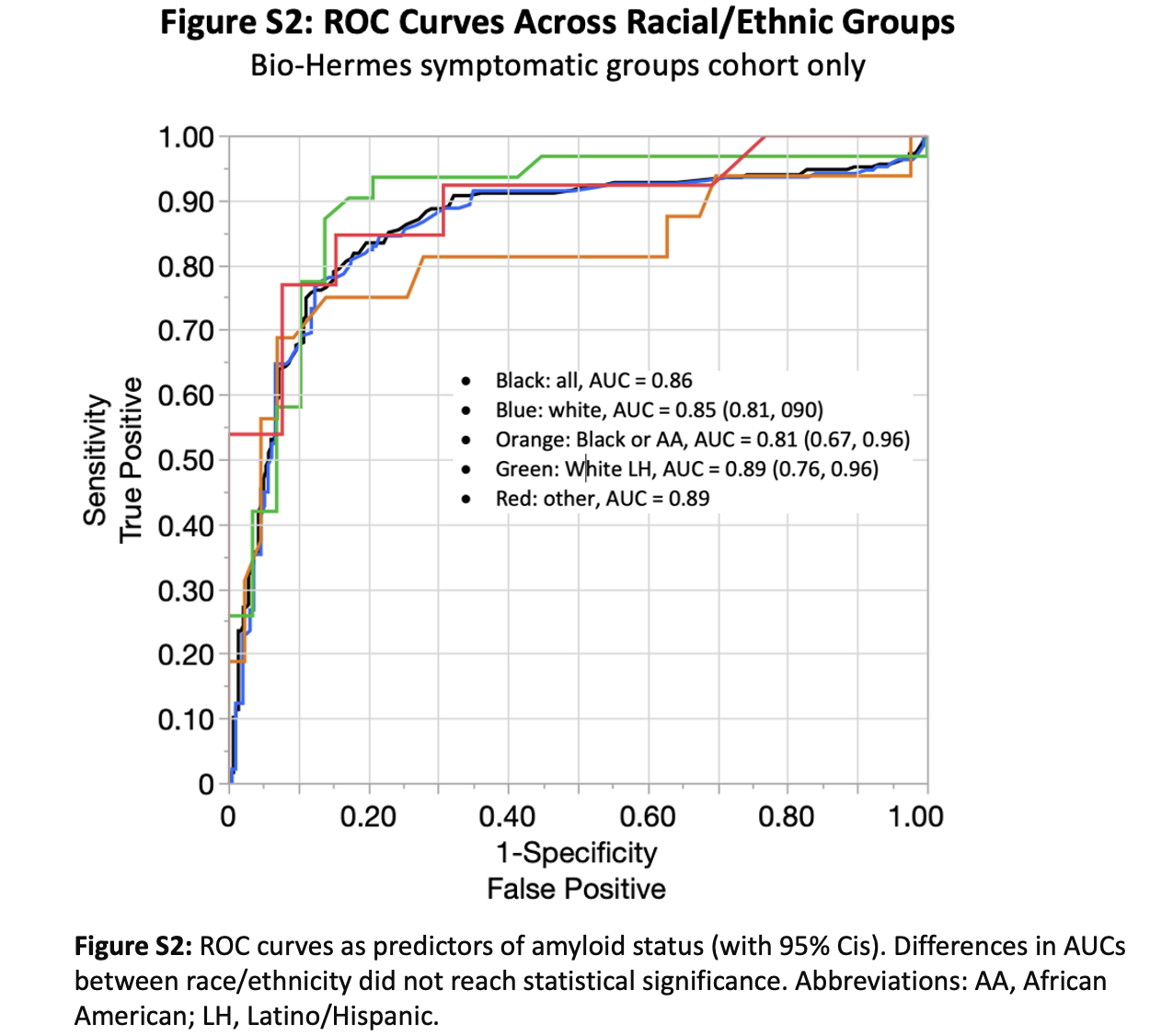


**Table S1: Demographic Characteristics by Cohort**


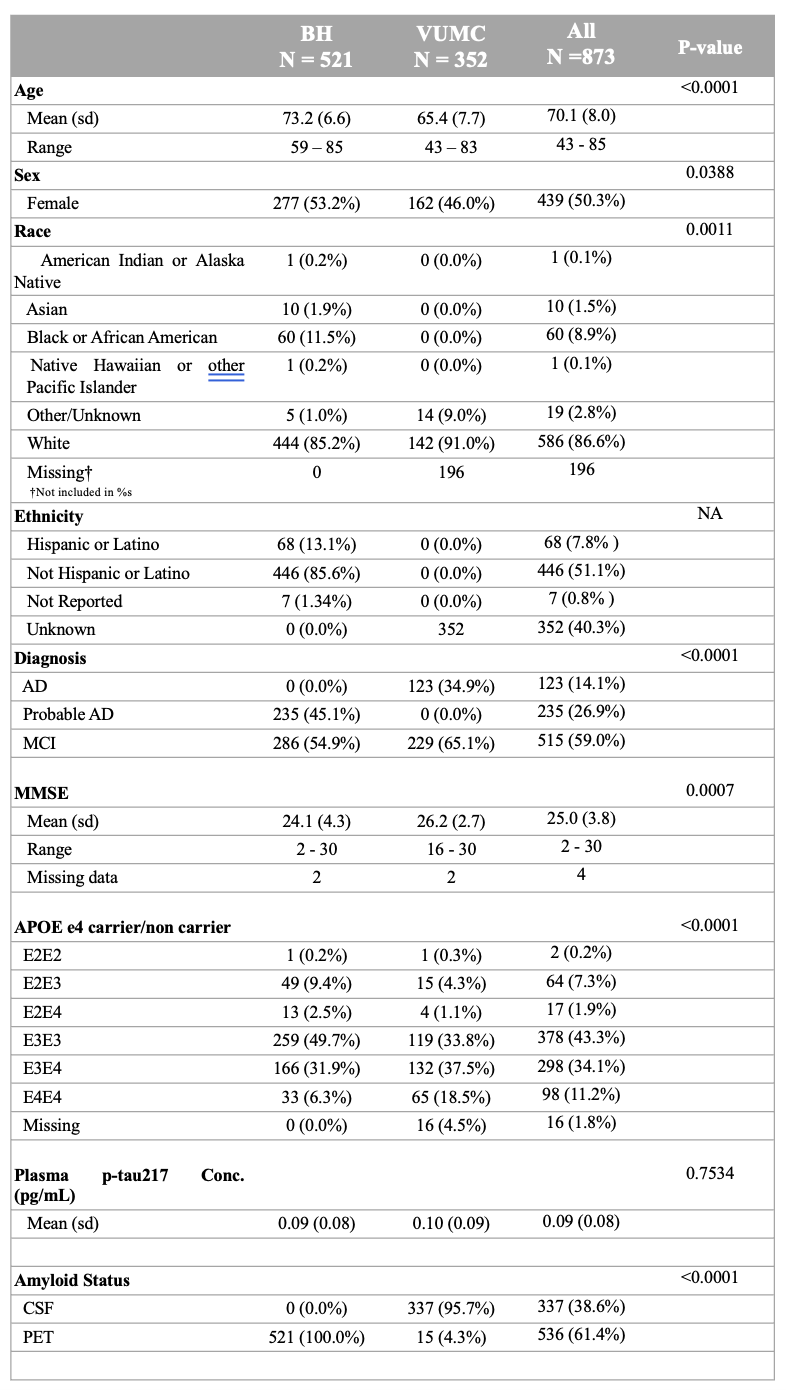


**Table S1**: Amyloid classification based on amyloid PET or CSF biomarkers. Abbreviations: MCI, mild cognitive impairment; MMSE, Mini-Mental State Examination; PET, positron emission tomography.


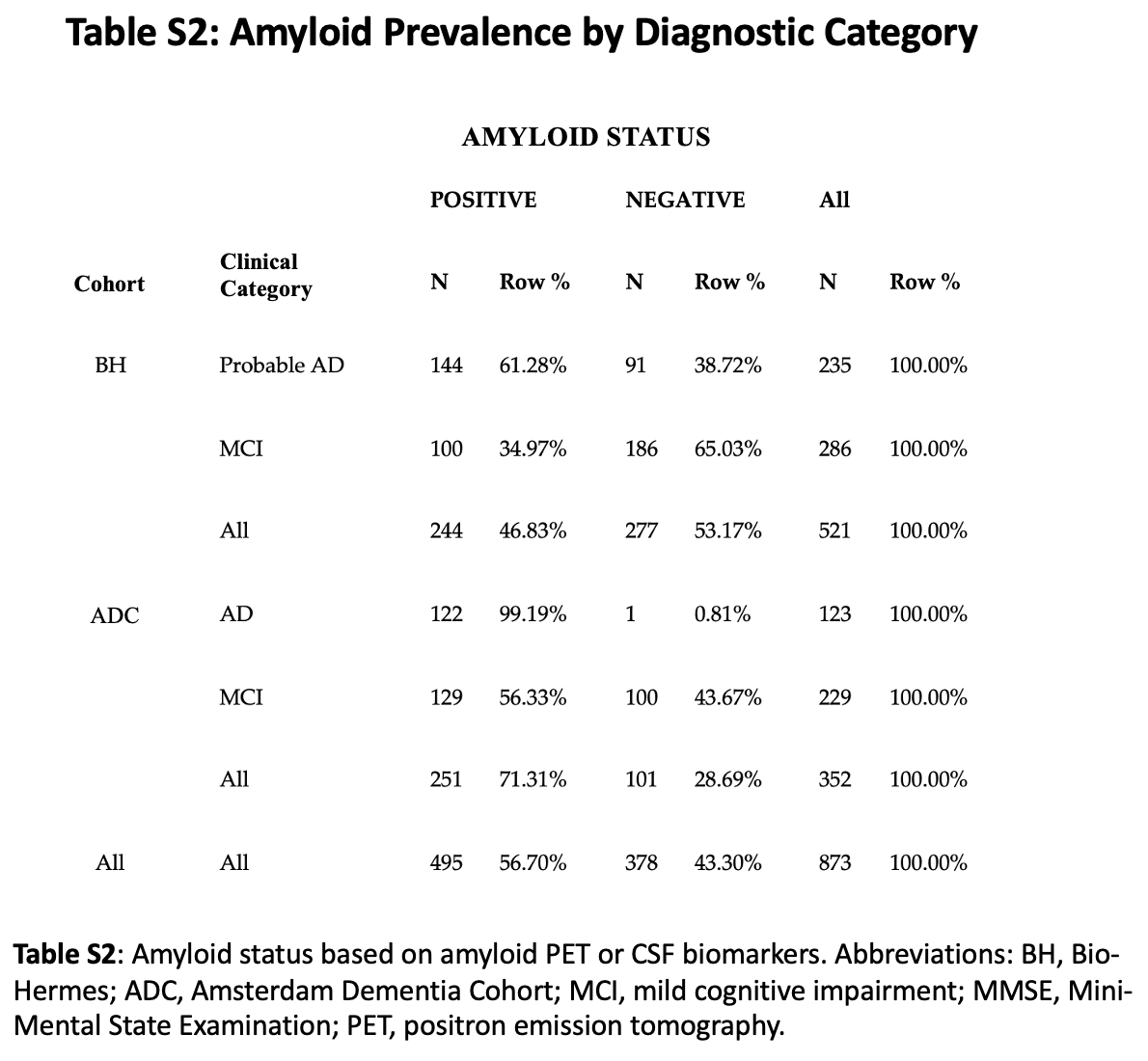


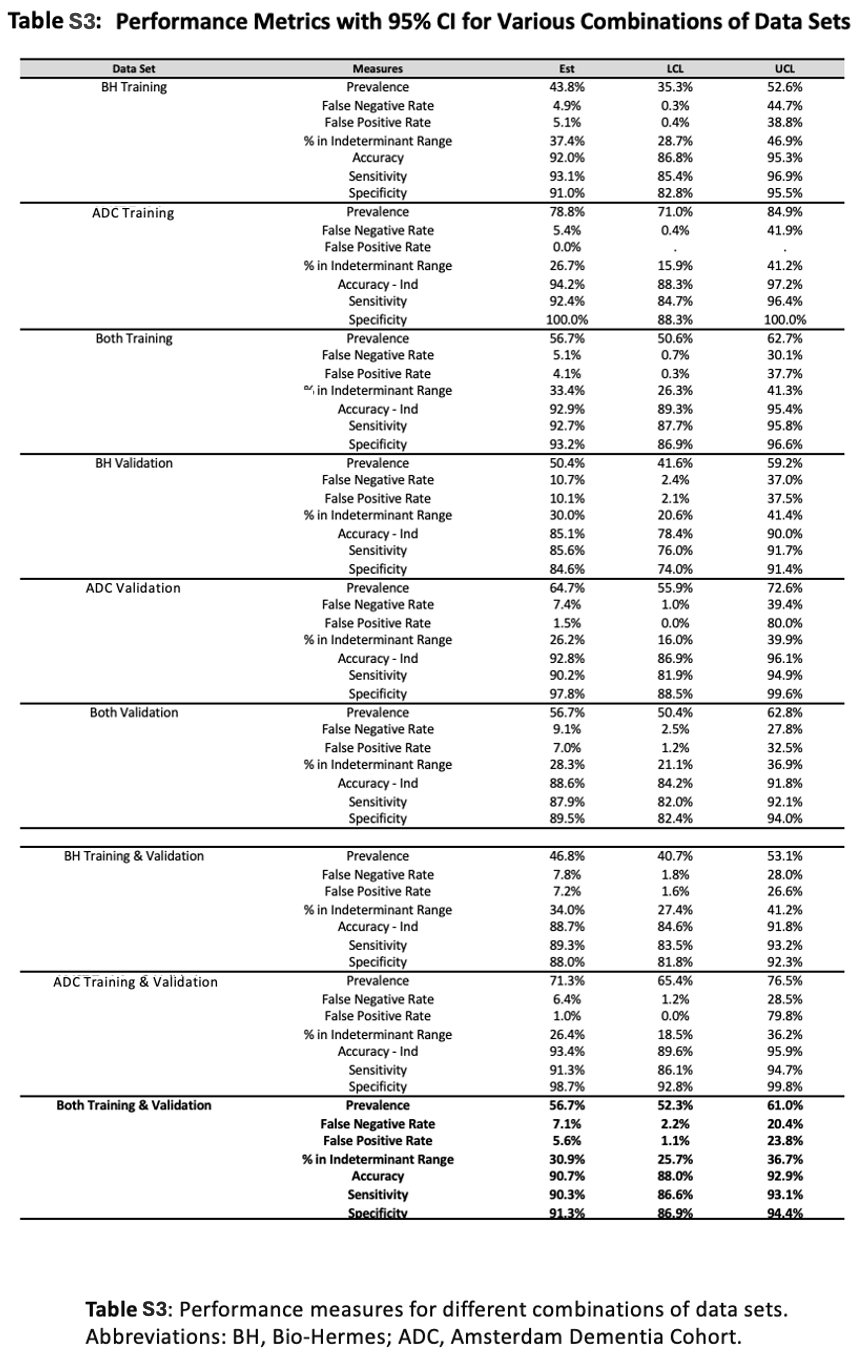


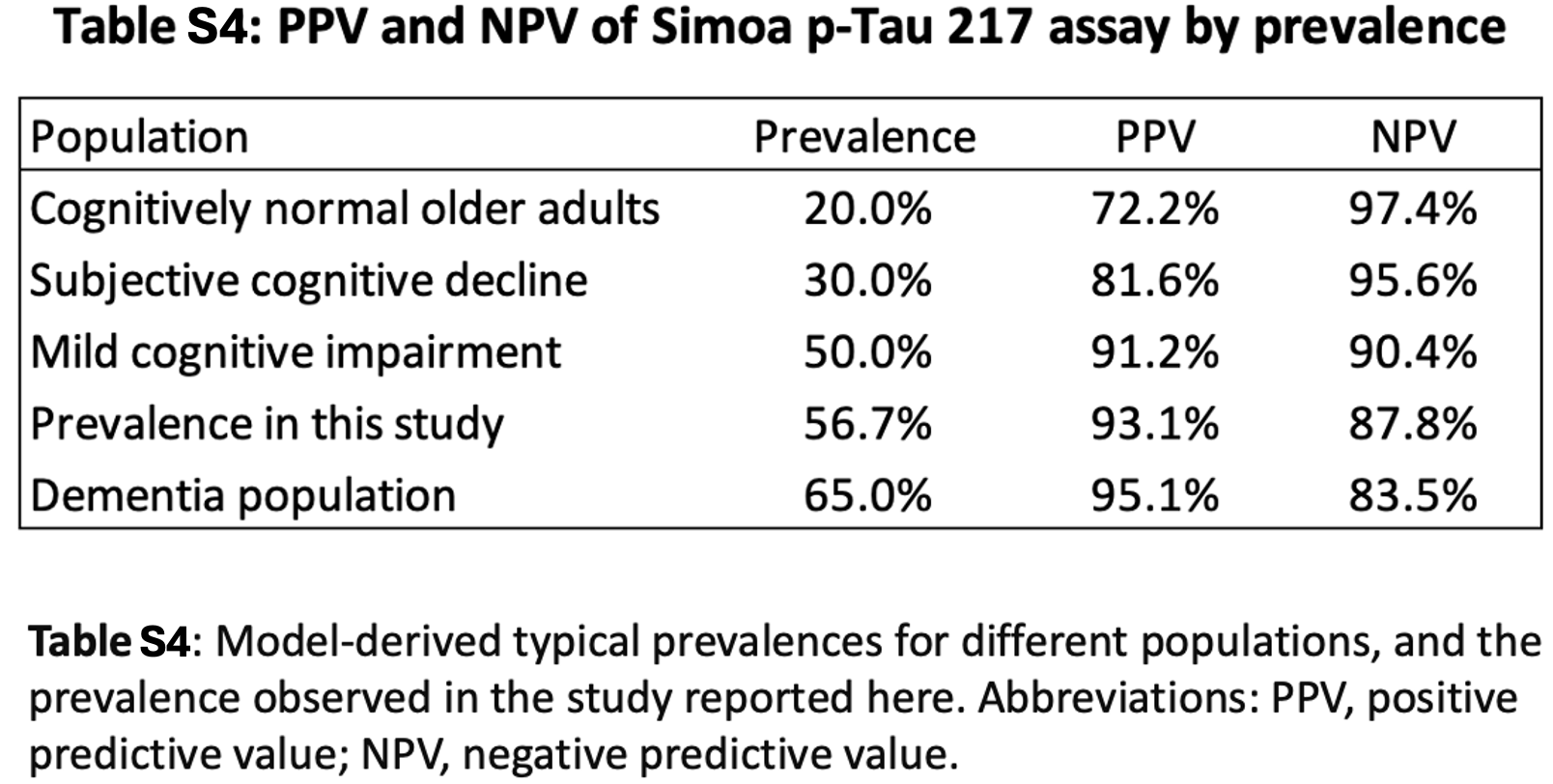


G. Supplemental Analyses: Accuracy of Simoa p-Tau 217 for Dementia with Lewy Bodies (DLB) and Frontal Temporal Dementia (FTD) Cases.

50 each of cases diagnosed with DLB and FTD were tested in the assay with optimized diagnostic thresholds. A proportion of these samples were also amyloid positive, and the accuracy of the test for detection of amyloid in these mixed pathology cases was characterized. Demographic and clinical characteristics of these samples are summarized in Table G1.

**Table G1: Demographic and clinical characteristics of DLB and FTD samples from the ADC**

|  | **DLB** | **FTD** |
| --- | --- | --- |
| n | 50 | 50 |
| Age (mean, SD) | 68.14 | 62.42 |
| Sex (male, %) | 41 (82.0) | 28 (56.0) |
| APOE carrier = yes (%) | 26 (53.1) | 12 (26.1) |
| MMSE (mean, SD) | 22.22 (4.84) | 24.29 (4.60) |
| CSF Abeta42 (mean, SD) | 772.22 (240.93) | 943.77 (280.47) |
| CSF p-Tau (mean, SD) | 51.72 (21.18) | 48.35 (21.89) |
| CSF Tau (mean, SD) | 377.46 (203.08) | 399.82 (234.98) |
| Amyloid positive by CSF (%) | 50% | 22% |

p-Tau 217 results compared with CSF amyloid status for the DLB and FTD cases are summarized in Tables G2 and G3 below.

Table G2: 2 x 3 Table for DLB cases

|  | | **p-Tau 217 Result** (Intermediate zone: 0.04 - 0.09 pg/mL) | | | | | | | |
| --- | --- | --- | --- | --- | --- | --- | --- | --- | --- |
|  | | **Low Risk** | | **Intermediate** | | **High Risk** | | **All** | |
| **Clinical population** | **Amyloid Status** | **N** | **Row %** | **N** | **Row %** | **N** | **Row %** | **N** | **Row %** |
| DLB | Positive | 0 | 0.0% | 20 | 80.0% | 5 | 20.0% | 25 | 100.0% |
|  | Negative | 12 | 48.0% | 10 | 40.0% | 3 | 12.0% | 25 | 100.0% |
|  | All | 12 | 24.0% | 30 | 60.0% | 8 | 16.0% | 50 | 100.0% |

Table G3: 2 x 3 Table for FTD cases

|  | | **p-Tau 217 Result** (Intermediate zone: 0.04 - 0.09 pg/mL) | | | | | | | |
| --- | --- | --- | --- | --- | --- | --- | --- | --- | --- |
|  | | **Low Risk** | | **Intermediate** | | **High Risk** | | **All** | |
| **Clinical population** | **Amyloid Status** | **N** | **Row %** | **N** | **Row %** | **N** | **Row %** | **N** | **Row %** |
| FTD | Positive | 2 | 18.2% | 4 | 36.4% | 5 | 45.5% | 11 | 100.0% |
|  | Negative | 23 | 59.0% | 14 | 35.9% | 2 | 5.12% | 39 | 100.0% |
|  | All | 25 | 50.0% | 18 | 36.0% | 7 | 14.0% | 50 | 100.0% |

Clinical performance metrics obtained for the DLB and FTD cases are summarized in Table G4:

Table G4: Performance metrics with 95% CI for DLB and FTD cases

| **Data Set** | **Measures** | **Est** | **LCL** | **UCL** |
| --- | --- | --- | --- | --- |
| DLB | Amyloid prevalence | 50.0% | 36.6% | 63.4% |
|  | False Negative Rate | 0.0% | 0.0% | 13.3% |
|  | False Positive Rate | 12.0% | 4.2% | 30.0% |
|  | % in intermediate zone | 60.0% | 46.2% | 72.4% |
|  | Accuracy (- Int) | 85.0% | 64.0% | 94.8% |
|  | Sensitivity (- Int) | 100.0% | 56.6% | 100.0% |
|  | Specificity (- Int) | 80.0% | 54.8% | 93.0% |
| FTD | Amyloid prevalence | 22.0% | 12.8% | 35.2% |
|  | False Negative Rate | 18.2% | 5.1% | 47.7% |
|  | False Positive Rate | 5.1% | 1.4% | 16.9% |
|  | % in intermediate zone | 36.0% | 24.1% | 49.9% |
|  | Accuracy (- Int) | 87.5% | 71.9% | 95.0% |
|  | Sensitivity (- Int) | 71.4% | 35.9% | 91.8% |
|  | Specificity (- Int) | 92.0% | 75.0% | 97.8% |

Despite the limited statistical powering from the small sampling sizes, the data suggest amyloid detection accuracy statistically consistent with the validation cohort for detecting amyloid in DLB and FTD cases. It is noted that although 100% sensitivity was observed in DLB cases, a majority (30) fell into the intermediate zone, suggesting a relatively weak amyloid signal.

The impact of inclusion of these non-AD and co-pathology cases into the validation cohort was assessed. Two different incidence levels were examined: “typical” percentages as reported in memory clinics, and “high” levels as might be encountered with under-diagnoses and among a younger population with high percentages of FTD. Table G5 summarizes the percentages that were included:

**Table G5: Percentage of DLB and FTD cases included in validation cohort**

| Dx category | Typical | High |
| --- | --- | --- |
| DLB | 4.6%^A1^ | 9.5% |
| FTD | 7.7%^A2-5^ | 9.5%^A-6^ |

The effect of the addition of up to 100 non-AD and AD co-pathology cases is depicted in Figure G1 below.

**Figure G1: Effect of the addition of DLB and FTD samples on performance metrics**


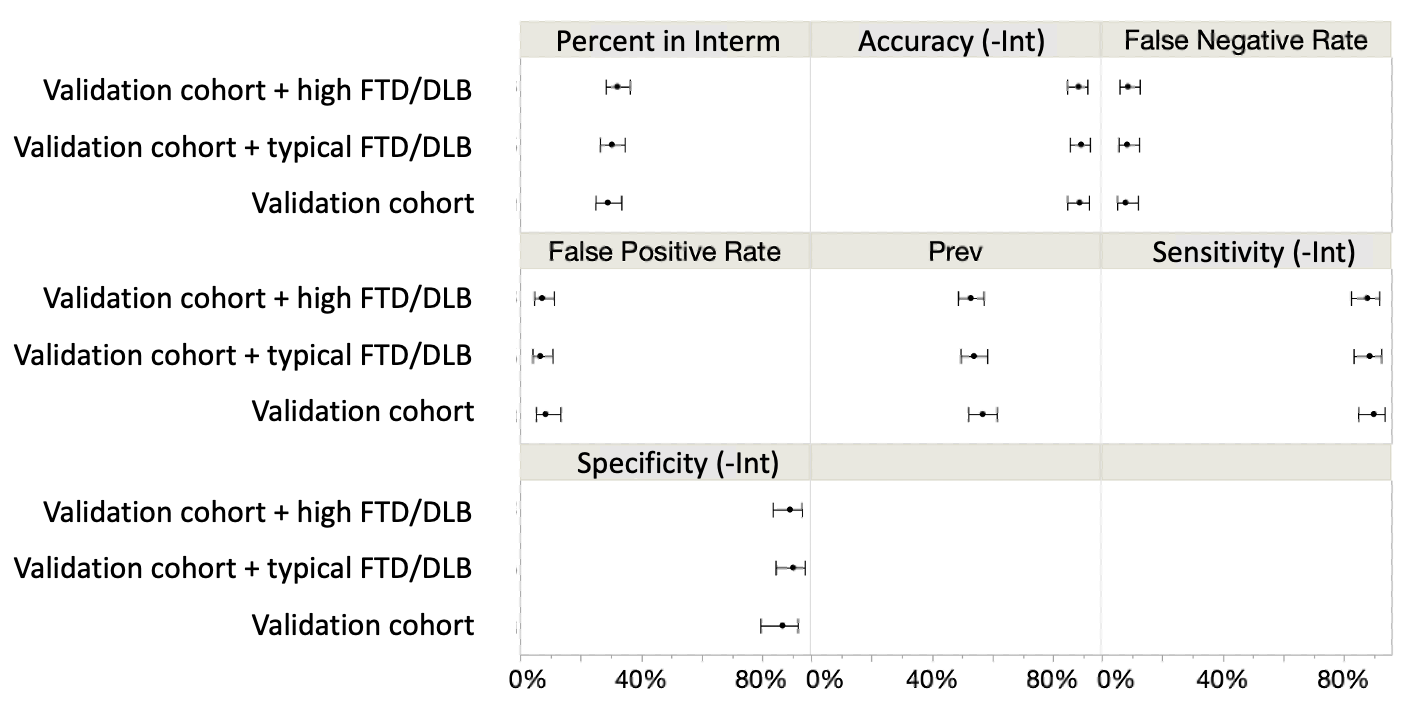


While the 30 DLB cases in the intermediate zone increased the overall validation cohort intermediate zone from 28% to 32%, there was no significant difference in the performance of the test in classifying amyloid status with up to 19% of non-AD and co-pathology cases added to the cohort.

**Supplemental References**

G1 Kane JPM, Surendranathan A, Bentley A, Barker SAH, Taylor JP, Thomas AJ, Allan LM, McNally RJ, James PW, McKeith IG, Burn DJ, O'Brien JT. Clinical prevalence of Lewy body dementia. Alzheimers Res Ther. 2018 Feb 15;10(1):19.

G2 Custodio N, Herrera-Perez E, Lira D, Montesinos R, Bendezu L. Prevalence of frontotemporal dementia in community-based studies in Latin America: a systematic review. Dement Neuropsychol. 2013 Jan-Mar;7(1):27-32.

G3 Rabinovici GD, Miller BL. Frontotemporal lobar degeneration: epidemiology, pathophysiology, diagnosis and management. CNS Drugs 2010; 24:375-398.

G4 Ratnavalli E, Brayne C, Dawson K, Hodges JR. The prevalence of frontotemporal dementia. Neurology 2002;58:1615-1621.

G5 Van Swieten JC, Rosso SM. Epidemiological aspects of frontotemporal dementia. Handb Clin Neurol 2008;89:331-341.

G6 van Gils AM, Rhodius-Meester HFM, Leeuwis AE, Handgraaf D, Bakker C, Peetoom K, Bouwman FH, Pijnenburg YAL, Papma JM, Hoogendoorn TA, Schoonenboom N, van Strien A, Verwey NA, Köhler S, de Vugt ME, van der Flier WM. Young-onset dementia in memory clinics in the Netherlands: Study design and description of PRECODE-GP. Alzheimers Dement (Amst). 2023 Aug 21;15(3):e12471.

**Table S5: Tukey-Kramer Comparisons of Pairwise Differences in Mean p-Tau 217 Values Across Racial/Ethnic Groups**

**Amyloid Positive Participants**


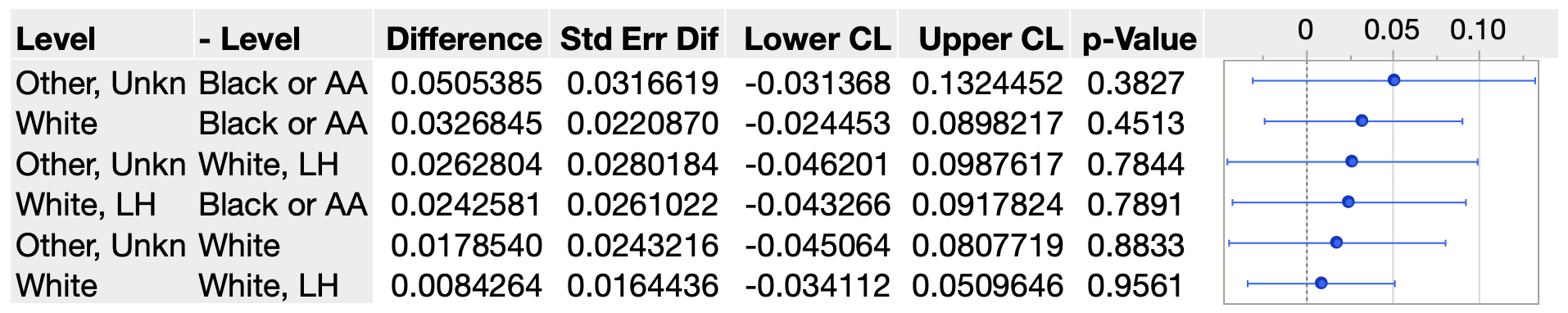


**Amyloid Negative Participants**


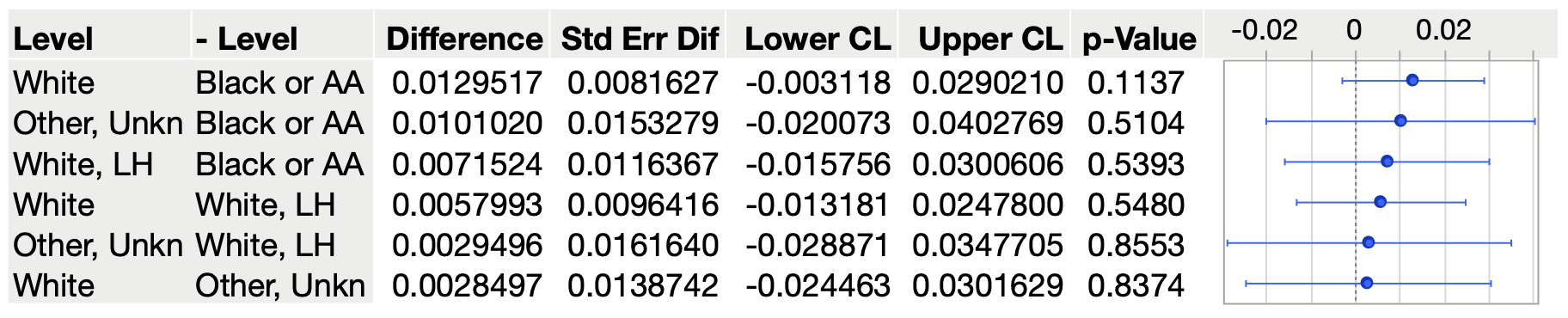


**Table S5:** Comparison of all R/E pairs using Tukey-Kramer multiple comparison. Differences in p-Tau 217 results were among the largest between white and black/AA groups, but these differences did not reach statistical significance for either the amyloid positive subjects (mean difference 0.051 pg/mL, p = 0.3827) or the amyloid negative subjects (mean difference 0.013 pg/mL, p = 0.1137).
